# When Genetics Meets the Mediterranean Diet Across Metabolic Diseases

**DOI:** 10.3390/nu18203348

**Published:** 2026-10-13

**Authors:** Francesca Gorini, Mariangela Palazzo, Morena Gabriele, Andrea Borghini

**Affiliations:** 1 Institute of Clinical Physiology, National Research Council, 56124 Pisa, Italy; mariangelapalazzo@cnr.it (M.P.); andrea.borghini@cnr.it (A.B.); 2 Institute of Agricultural Biology and Biotechnology, National Research Council, 56124 Pisa, Italy; morena.gabriele@ibba.cnr.it

**Keywords:** Mediterranean diet, nutrigenetics, gene–diet interaction, type 2 diabetes, obesity, metabolic syndrome, MASLD, genetic susceptibility, genetic risk score, precision nutrition

## Abstract

Metabolic diseases and related cardiometabolic conditions, including type 2 diabetes (T2D), obesity, metabolic syndrome (MetS), and metabolic dysfunction-associated steatotic liver disease (MASLD), arise from complex interactions between genetic predisposition and environmental factors. Among dietary patterns, the Mediterranean diet (MedDiet) has consistently been associated with improved metabolic health and a reduced risk of cardiometabolic diseases. However, substantial interindividual variability in metabolic responses to dietary exposures suggests that genetic susceptibility may contribute to differences in response to the MedDiet. Over the past two decades, nutrigenetic research has investigated whether adherence to the MedDiet can modify the effects of genetic variants associated with metabolic risk. Initial studies focused primarily on candidate genes involved in glucose metabolism, adiposity regulation, circadian rhythms, and hepatic lipid metabolism, including variants in genes such as *TCF7L2*, *FTO*, *CLOCK*, *ADIPOQ*, and *PNPLA3*. More recently, research has expanded to genetic risk score and polygenic risk score approaches, providing a broader assessment of inherited susceptibility. This literature review summarizes current evidence on MedDiet–gene interactions in T2D, obesity, MetS, MASLD, and related cardiometabolic traits, highlighting the transition from candidate-gene approaches to aggregate genetic risk models. Overall, the available literature suggests that the beneficial effects of the MedDiet are observed across different levels of genetic susceptibility, whereas evidence supporting clinically relevant gene–diet interactions remains heterogeneous and limited. Further large-scale, multi-ancestry, and well-designed prospective studies are needed before genetically tailored MedDiet recommendations can be routinely implemented in clinical practice.

## 1. Introduction

Metabolic diseases, together with related cardiometabolic conditions such as metabolic syndrome (MetS), encompass a spectrum of complex disorders including obesity, type 2 diabetes (T2D), and metabolic dysfunction-associated steatotic liver disease (MASLD). These conditions are characterized by interconnected dysregulated processes, including obesity-related insulin resistance, dyslipidemia, dysglycemia, hypertension, and chronic low-grade systemic inflammation [1,2,3], and are associated with increased risks of disability, cardiovascular disease (CVD), cancer, and premature mortality [1,4]. Recent estimates indicate that the burden of most metabolic diseases and related risk factors has increased steadily over the past three decades, with the fastest growth observed for T2D and obesity [4].

Although aging, microbiome composition, unhealthy lifestyle behaviors, and other environmental factors are established determinants of metabolic disease development, genetic susceptibility also plays a pivotal role in shaping interindividual variability in disease risk, progression, and clinical outcomes, ultimately influencing both prognosis and the effectiveness of preventive and therapeutic strategies [5,6,7]. In parallel, advances in high-throughput genotyping technologies, coupled with an improved understanding of the human genome, have transformed the investigation of the genetic basis of complex diseases [8]. Genome-wide association studies (GWASs) and whole-exome sequencing have identified hundreds of loci associated with obesity, T2D, MASLD, and related cardiometabolic traits [8,9], involving pathways linked to glucose homeostasis [10,11,12], adiposity [13,14], and hepatic lipid metabolism [15,16], while emerging evidence has also implicated circadian regulation in metabolic disease susceptibility [17,18,19].

Despite the growing number of genetic variants associated with metabolic traits, the effect sizes of individual variants are generally modest, with most explaining only a small proportion of phenotypic variance or disease susceptibility. This highlights the substantial contribution of environmental and lifestyle-related factors, as well as gene–environment interactions, to disease development and progression [20,21,22]. Among these modifiers, dietary patterns have emerged as key determinants of metabolic health and important potential modulators of genetic susceptibility [23,24]. Accordingly, nutrigenetics and nutrigenomics have become important areas of research in personalized nutrition, investigating, respectively, how genetic variation influences responses to dietary factors and how nutrients and dietary patterns modulate gene expression and metabolic pathways [9,25].

The Mediterranean diet (MedDiet) is recognized as one of the healthiest dietary patterns, with its cardiometabolic benefits attributed to a combination of lipid-lowering, anti-inflammatory, antioxidant, and insulin-sensitizing effects [26,27]. Findings from human studies suggest that adherence to the MedDiet may influence the relationship between genetic susceptibility and metabolic health outcomes, supporting the concept that dietary patterns can partly modulate the phenotypic expression of inherited risk, e.g., [15,28,29,30]. While much of the available evidence has focused on individual genetic variants, recent advances in genetic epidemiology have led to the development of genetic risk scores (GRSs) and polygenic models that combine the effects of multiple susceptibility loci, providing a more comprehensive estimate of inherited susceptibility and opening new avenues for precision dietary strategies, e.g., [31,32,33].

Therefore, this review summarizes current evidence on the role of the MedDiet as a modifier of genetic susceptibility to metabolic diseases and related cardiometabolic conditions. Particular attention is devoted to human studies investigating gene–diet interactions in relation to obesity, T2D, MetS, MASLD, and cardiometabolic traits, as well as the transition from candidate-gene studies to genetic risk scores and polygenic approaches in precision nutrition.

## 2. Literature Search Strategy

This review was informed by a targeted literature search of PubMed, conducted in July 2026 and covering studies published up to the date of the search. The search focused on three main concepts: the MedDiet and Mediterranean dietary patterns; genetic susceptibility, including individual genetic variants and aggregate measures of genetic risk; and metabolic traits and diseases. Search terms included, among others, “Mediterranean diet,” “Mediterranean dietary pattern,” “Mediterranean diet score,” “Mediterranean diet adherence,” “Mediterranean-style diet,” “genetic variant,” “SNP,” “polymorphism,” “genetic risk score,” “polygenic risk score,” “polygenic score,” “gene–diet interaction,” “nutrigenetics,” “nutrigenomics,” “obesity,” “type 2 diabetes,” “metabolic syndrome,” “MASLD,” “insulin resistance,” “adiposity,” “body mass index,” “body weight,” “lipid profile,” and “hepatic steatosis”.

To capture studies investigating specific dietary features of the Mediterranean dietary pattern, the search also included terms related to major dietary components and characteristics of Mediterranean-style interventions, including “extra virgin olive oil,” “virgin olive oil,” “olive oil,” “monounsaturated fatty acids,” “MUFA,” “nuts,” and “Mediterranean diet intervention.” Studies examining individual Mediterranean diet components or specific aspects of dietary fat composition were considered when they provided relevant evidence on gene–diet interactions or genotype-dependent metabolic responses that could contribute to the understanding of the broader Mediterranean dietary pattern.

Studies were included if they: (i) investigated interactions between genetic variants, genetic risk scores, or other measures of genetic susceptibility and adherence to the Mediterranean dietary pattern or its major dietary components and (ii) evaluated metabolic traits or metabolic disease outcomes, including obesity, T2D, MetS, MASLD, adiposity-related traits, insulin resistance, or lipid-related outcomes. Studies focusing exclusively on genetic or dietary associations without an interaction component were considered only when relevant for describing the genetic background of the metabolic conditions or for contextualizing the evidence discussed in the review. Studies were excluded if they did not assess a dietary exposure or were conference abstracts, editorials, or commentaries without original data.

The search was not restricted by study design or population, and both observational and interventional studies were considered. Studies were selected according to their relevance to the scope and objectives of the review, with particular emphasis on human evidence addressing interactions between genetic variation or aggregate genetic risk and the MedDiet, Mediterranean-style dietary patterns, or their major components in relation to metabolic traits, metabolic diseases, and related cardiometabolic conditions.

Relevant systematic reviews, meta-analyses, and narrative reviews were also examined to identify additional relevant studies through reference-list screening. These secondary sources were used to identify potentially relevant primary studies rather than as primary sources of evidence. The identified evidence was organized according to the genetic approach adopted, distinguishing studies investigating individual genetic variants from those evaluating aggregate genetic risk using genetic risk scores.

The search was designed as a targeted literature search to identify and synthesize relevant evidence within the scope of this review and was not intended to provide a systematic or exhaustive assessment of all published studies.

## 3. Genetic Architecture of Metabolic Diseases

Twin and family studies have consistently demonstrated that metabolic disorders are partially heritable, although estimates vary across conditions, ranging from approximately 40–70% for obesity [34] to up to 72% for T2D [35,36], 27–64% for MetS [37], and 30–60% for MASLD [38]. Building on these findings, GWASs have advanced the study of inherited susceptibility by enabling the genome-wide analysis of hundreds of thousands to millions of single-nucleotide polymorphisms (SNPs) and the identification of numerous loci associated with metabolic traits and diseases [39,40]. This represented a major advance over the traditional candidate-gene approach, in which genes are selected based on prior knowledge of their biological role and potential involvement in the phenotype [39,41]. The following sections summarize representative susceptibility genes and variants implicated in metabolic diseases, highlighting their contribution to disease risk across different metabolic phenotypes.

### 3.1. Genetic Determinants of Type 2 Diabetes

T2D is a complex and heterogeneous metabolic disorder characterized by progressive, nonautoimmune β-cell dysfunction and insulin secretion decline, frequently occurring in the presence of insulin resistance and MetS [42]. Accounting for approximately 90–95% of diabetes cases worldwide, T2D is primarily manifested by chronic hyperglycemia [43]. Its common polygenic form reflects the interplay between impaired insulin secretion, insulin resistance, and genetic susceptibility, with numerous variants contributing to interindividual differences in disease risk [44]. The genetic architecture of T2D has been progressively elucidated through candidate-gene studies, linkage analyses, GWASs, and sequencing approaches [45]. Recent multi-ancestry GWAS analyses have identified 1289 independent association signals mapping to 611 T2D-associated loci, highlighting the genetic heterogeneity of T2D and implicating pathways related to β-cell dysfunction, insulin resistance, and cardiometabolic complications [46].

Among the susceptibility loci identified to date, transcription factor 7-like 2 (TCF7L2), located on chromosome 10q25.3 and encoding a member of the T-cell factor/lymphoid enhancer factor (TCF/LEF) family, is one of the strongest genetic determinants of T2D risk [45,47]. The rs7903146 T risk allele has been consistently associated with T2D across populations of diverse ancestries, conferring an approximately 1.4-fold increased risk [48,49]. Although the underlying mechanisms are not fully elucidated, TCF7L2 is a key downstream effector of the canonical Wnt/β-catenin pathway and regulates proglucagon expression in enteroendocrine cells, as well as transcriptional programs involved in β-cell function, insulin secretion, glucose homeostasis, adipocyte biology, and energy metabolism [47,50]. Altered TCF7L2 activity may also affect adipose tissue function, contributing to insulin resistance and glucose intolerance [51]. Other variants, including rs12255372, rs114770437, and rs11196205, have also been implicated in T2D susceptibility [52,53,54,55,56,57], although rs7903146 remains the most extensively replicated and functionally characterized variant.

The *SLC30A8* gene encodes zinc transporter 8 (ZnT8), a protein predominantly expressed in pancreatic β-cell secretory granules and involved in zinc transport and insulin storage. It was among the first T2D susceptibility genes identified through GWASs [58]. The common missense variant rs13266634 (R325W), with the C allele associated with increased T2D risk, is one of the most extensively studied coding variants at this locus [58,59]. The C risk allele encodes the Arg325 rather than the Trp325 isoform and has been proposed to affect ZnT8 function, potentially altering zinc transport and insulin processing and secretion [45,59,60]. Consistent with this role, individuals homozygous for the C risk allele exhibit a 19% reduction in first-phase insulin secretion following an intravenous glucose challenge [61]. Large meta-analyses further show that the C allele is associated with a 14–23% increased risk of T2D across diverse populations, supporting its consistent contribution to T2D susceptibility across ancestries [62,63].

**Figure 1:**
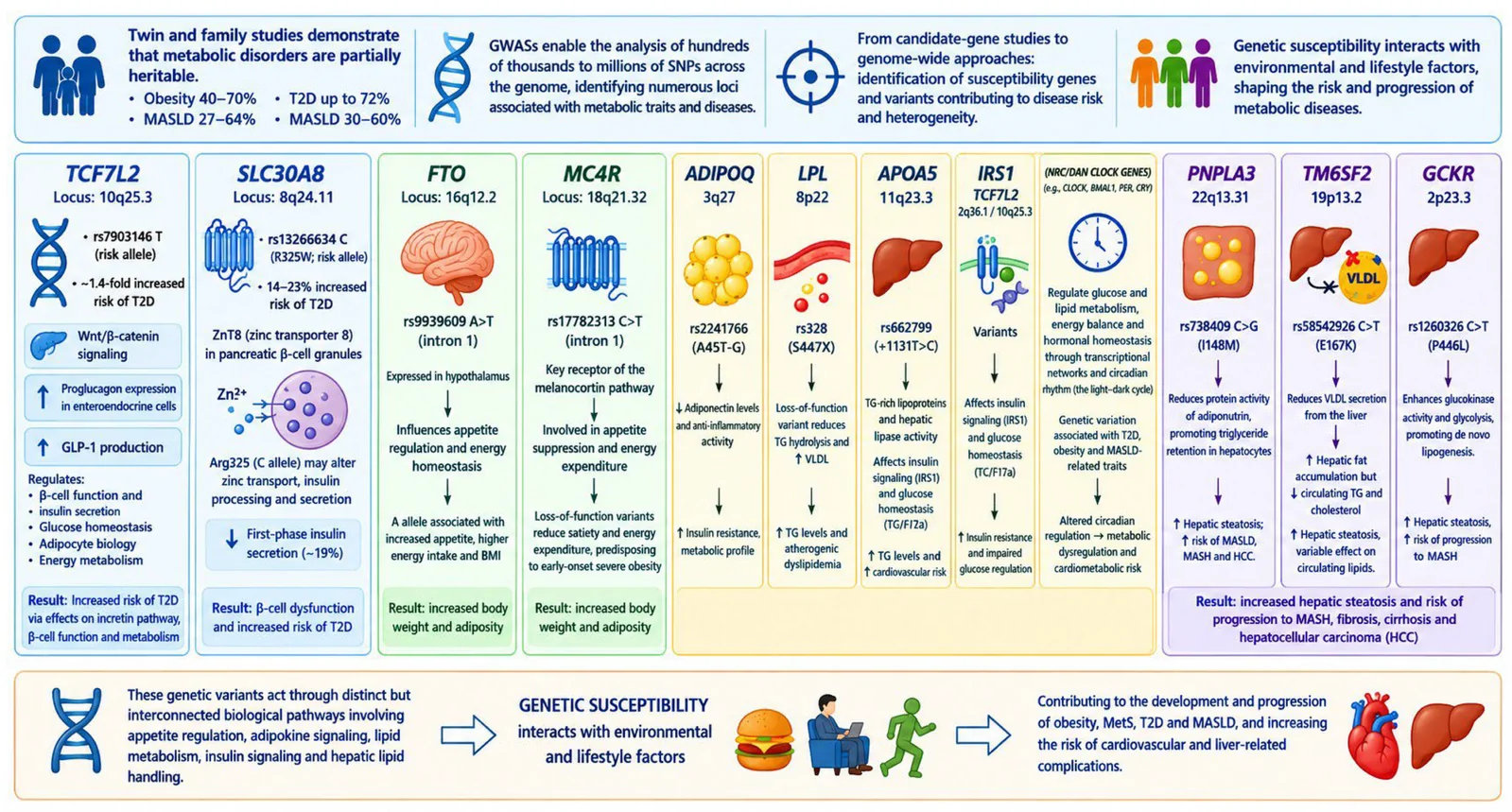
Genetic architecture of metabolic diseases. Overview of the main genetic determinants and biological pathways contributing to susceptibility to type 2 diabetes (T2D), obesity, metabolic syndrome (MetS), and metabolic dysfunction-associated steatotic liver disease (MASLD). The figure highlights representative susceptibility genes and variants, including *TCF7L2* and *SLC30A8* for T2D; *FTO* and *MC4R* for obesity and adiposity; *ADIPOQ*, *LPL*, *APOA5*, and *IRS1* for MetS and cardiometabolic traits; and *PNPLA3*, *TM6SF2*, and *GCKR* for MASLD. Circadian clock genes, including *CLOCK*, *BMAL1* (*ARNTL*), *PER*, and *CRY*, are also shown as an additional layer of metabolic genetic susceptibility through their role in regulating glucose and lipid metabolism, energy balance, and hormonal homeostasis. Collectively, these genetic determinants influence diverse biological processes, including β-cell function and insulin secretion, appetite regulation and energy homeostasis, adipose tissue function, lipid metabolism, insulin signaling, and hepatic lipid accumulation, while interacting with environmental and lifestyle factors to shape interindividual differences in metabolic phenotypes and disease risk. Image created with the assistance of ChatGPT (OpenAI, GPT-5.5).

The *PPARG* gene encodes peroxisome proliferator-activated receptor gamma (PPARγ), a ligand-activated nuclear receptor that regulates adipocyte differentiation, lipid metabolism, insulin sensitivity, and inflammatory responses [63]. Given its central role in metabolic homeostasis, *PPARG* is one of the most extensively studied candidate genes for T2D susceptibility and metabolic traits [64]. Although predominantly expressed in adipose tissue, PPARγ is also expressed in pancreatic β-cells, where it contributes to β-cell function and lipid metabolism [45,65]. The Pro12Ala polymorphism is the most extensively studied *PPARG* variant and results in a proline-to-alanine substitution at codon 12 of the PPARγ2 isoform, potentially affecting transcriptional activity, adipose tissue function, and glucose homeostasis [65,66]. Meta-analyses suggest that the Ala12 allele is associated with a modest reduction in T2D risk compared with the Pro12 allele, although the magnitude of the association varies across populations and genetic models [64,67,68] (Figure 1).

Overall, these findings highlight the polygenic architecture of T2D, with multiple variants affecting β-cell function, insulin secretion, and insulin action contributing to disease susceptibility. The modest effects of individual variants underscore the importance of considering the cumulative contribution of multiple risk loci when assessing genetic predisposition to T2D.

### 3.2. Genetic Determinants of Obesity and Adiposity

Obesity is a complex, multifactorial chronic disease associated with an increased risk of numerous comorbidities, including T2D, CVD, and several cancers [69]. Although sustained positive energy balance is required for weight gain, susceptibility to obesity is strongly influenced by genetic predisposition, together with epigenetic, environmental, behavioral, and sociocultural factors [70]. While rare monogenic and syndromic forms result from pathogenic variants in single genes or chromosomal abnormalities affecting energy homeostasis, most obesity is polygenic, reflecting the cumulative and largely additive effects of numerous common variants, each conferring a modest increase in risk [70,71].

GWASs have identified hundreds of loci associated with bodey mass index (BMI) and obesity, highlighting the highly polygenic architecture of common obesity [70]. Analyses of 97 BMI-associated loci showed significant enrichment in genes expressed in the hypothalamus and pituitary gland, key regions involved in appetite regulation, as well as in the hippocampus and limbic system, suggesting a major contribution of neural pathways to body weight regulation [72]. Among these loci, variants in the fat mass and obesity-associated (FTO) and melanocortin-4 receptor (MC4R) genes represent the most consistently replicated genetic determinants of obesity susceptibility.

*FTO*, a large gene located on chromosome 16q12.2 and expressed in multiple tissues, including the hypothalamus, encodes a Fe(II)- and 2-oxoglutarate-dependent dioxygenase involved in RNA and nucleic acid demethylation and implicated in energy homeostasis and adipose tissue biology [70,73,74]. Among *FTO* variants, rs9939609 and rs1421085, both located within the first intron, are the most extensively studied and have been strongly associated with obesity-related traits [75,76]. Carriers of the rs9939609 A allele (AA/AT) and rs1421085 C allele (CC/CT) have been associated with obesity susceptibility and unfavorable metabolic profiles, including altered HDL cholesterol and TG levels in children and adolescents [76]. Each additional *FTO* risk allele has been associated with an approximately 1–1.5 kg higher body weight and a 20–30% increased risk of obesity [77]. Meta-analyses suggest that the strength of the associations between rs9939609 or rs1421085 and obesity may vary across populations and genetic models, with rs9939609 showing a slightly stronger association in Asian than Western populations and rs1421085 yielding ORs of 1.24–1.50 across different genetic models [78,79]. *FTO* variants have also been associated with T2D, although this association is substantially attenuated after adjustment for BMI, suggesting that increased adiposity contributes substantially to the observed relationship [80].

*MC4R*, located on chromosome 18q21 and encoding a G protein-coupled receptor expressed predominantly in the hypothalamus and also detected in adipocytes, is a key component of the central melanocortin system involved in nutrient sensing, satiety regulation, and metabolic responses [70,77,81]. It contributes to the regulation of food intake and energy expenditure [77]. More than 300 *MC4R* variants have been identified, with loss-of-function variants representing the most common cause of monogenic obesity and accounting for up to 6% of severe early-onset cases [70,82]. Common variants at the *MC4R* locus have also been investigated in relation to obesity [83]. Among these, rs17782313, located downstream of *MC4R*, is the most extensively studied, with the risk C allele associated with higher BMI and increased obesity risk [83]. Meta-analyses have confirmed this association, reporting ORs of 1.32–1.93 across genetic models and higher BMI and waist circumference among C allele carriers [81,84]. Although the underlying mechanisms remain incompletely understood, risk-allele carriers have been reported to exhibit greater appetite despite no significant differences in reported energy intake, suggesting altered appetite regulation as a potential contributor to obesity susceptibility [85]. The rs17782313 polymorphism has also been associated with T2D risk, independently of BMI [86] (Figure 1).

Collectively, common obesity is a highly polygenic disorder in which numerous variants involved in appetite regulation, energy homeostasis, and adipose tissue biology contribute to interindividual differences in susceptibility. Importantly, the phenotypic effects of these variants are not deterministic but are modulated by environmental and lifestyle factors, including dietary patterns and physical activity.

### 3.3. Genetic Determinants of Metabolic Syndrome and Cardiometabolic-Related Traits

MetS is a heterogeneous, multifactorial disorder characterized by the clustering of cardiometabolic abnormalities, including central obesity, atherogenic dyslipidemia, elevated blood pressure, and impaired glucose regulation [87]. It is associated with up to a fivefold increased risk of T2D, a 1.5-fold increased risk of cardiovascular events, and an approximately threefold higher risk of all-cause mortality [88,89]. Although lifestyle factors, including diet, physical activity, smoking, alcohol consumption, and sleep, play a central role in MetS development, marked interindividual variability in its clinical presentation and familial clustering reflect a complex interplay between genetic susceptibility and environmental influences, supporting its polygenic basis [87,90].

Consistent with its heterogeneous clinical presentation, the genetic architecture of MetS is complex and reflects the contribution of multiple variants involved in adiposity, insulin signaling, lipid metabolism, inflammation, and cardiovascular homeostasis [91]. Among genes implicated in MetS susceptibility, *ADIPOQ*, located on chromosome 3q27 and encoding the adipokine adiponectin, is one of the most extensively studied candidate genes [92]. Predominantly secreted by adipose tissue, adiponectin contributes to metabolic and cardiovascular homeostasis and regulates inflammatory processes, with insulin-sensitizing, anti-inflammatory, and anti-atherogenic effects [92,93]. Accordingly, reduced circulating adiponectin levels have been associated with insulin resistance, dyslipidemia, and increased susceptibility to MetS-related traits, including central obesity, T2D, hypertension, and CVD [92]. Numerous *ADIPOQ* variants have therefore been investigated in relation to MetS and its individual components, with rs2241766 (+45T>G) and rs1501299 (+276G>T) among the most extensively studied [94,95]. Compared with the TT genotype, the TG and GG genotypes of rs2241766 were associated with increased MetS risk (odds ratio—OR = 1.3 and 2.5, respectively), while the GT and TT genotypes of rs1501299 were associated with ORs of 1.5 and 2.3, respectively [95]. rs2241766, located in exon 2 near the exon–intron boundary, may influence *ADIPOQ* expression, whereas rs1501299 is an intronic variant with no clearly established biological function [95]. Meta-analyses have also reported associations between *ADIPOQ* variants and T2D and obesity, supporting a potential contribution of variation at this locus to multiple metabolic phenotypes [96,97].

*LPL*, located on chromosome 8p22, encodes lipoprotein lipase, a triglyceride lipase expressed in several tissues, including adipose tissue, skeletal muscle, heart, lung, and brain [98,99]. LPL is a key enzyme in lipid metabolism that hydrolyzes triglycerides (TG) in circulating chylomicrons and very-low-density lipoproteins (VLDL), promoting the release of free fatty acids for uptake by peripheral tissues [100]. Hundreds of coding and non-coding *LPL* variants have been identified, many of which have been associated with complex diseases, including obesity and coronary heart disease [98,101,102]. Genetic variation affecting LPL activity may alter lipid hydrolysis, contributing to increased plasma TG and reduced high-density lipoprotein (HDL) cholesterol levels [101]. Notably, the Ser447Ter (S447X) variant in exon 9, which results in premature termination of the protein two amino acids before its normal C-terminus, has been inversely associated with MetS [99,103]. Female non-carriers had a significantly higher likelihood of developing MetS (OR = 1.46), while another large study reported a 37% reduction in relative MetS risk among S447X carriers, together with a more favorable lipid profile but no significant associations with waist circumference (WC), hypertension, or glucose intolerance [99,103]. Beyond MetS, a meta-analysis also reported an association between S447X and reduced T2D risk [104].

Apolipoprotein A-V (apoA-V), encoded by the *APOA5* locus on chromosome 11q23, plays an important role in regulating plasma TG and HDL cholesterol levels [105,106]. APOA5 binds the angiopoietin-like protein 3/8 (ANGPTL3/8) complex and attenuates its inhibition of LPL activity, thereby promoting triglyceride-rich lipoprotein clearance, whereas APOA5 deficiency enhances ANGPTL3/8-mediated inhibition of LPL and impairs triglyceride clearance, resulting in elevated plasma TG levels [10,107]. Several *APOA5* polymorphisms have been associated with MetS components, particularly TG levels. The rs662799 (−1131T>C) variant has been consistently associated with higher TG concentrations, with the C allele conferring an unfavorable lipid profile [106,108,109]. Meta-analyses further confirmed an association between the −1131C allele and MetS, with carriers showing a 32–43% higher risk of developing the syndrome across different genetic models [106,108,110].

In addition to genes involved in lipid metabolism, genes related to insulin signaling and glucose homeostasis, including *IRS1* and *TCF7L2*, have also been implicated in metabolic phenotypes related to MetS [110,111,112]. *IRS1* encodes insulin receptor substrate 1, a key adaptor protein in the insulin signaling pathway, and the rs2943641 variant has been associated with insulin resistance and hyperinsulinemia and shown to modulate MetS reversion in response to a weight-loss dietary intervention [110,111]. *TCF7L2* polymorphisms have also been linked to context-dependent susceptibility to MetS, T2D, and dyslipidemia [112].

Furthermore, growing evidence suggests that circadian regulatory pathways may contribute to metabolic disease susceptibility. Circadian clock genes, including *CLOCK*, *BMAL1* (*ARNTL*), *PER*, and *CRY*, regulate glucose and lipid metabolism, energy balance, and hormonal homeostasis through transcriptional networks that coordinate metabolic processes with the light–dark cycle [18,19]. The circadian clock also plays an important role in the function of peripheral tissues, including the liver, pancreas, and adipose tissue, where circadian regulation contributes to glucose and lipid homeostasis and insulin sensitivity [113,114,115]. Disruption of circadian rhythms may impair metabolic homeostasis and contribute to metabolic dysfunction, including impaired glucose and lipid metabolism, insulin resistance, and increased risk of obesity and T2D [113,114,115]. In the liver, disruption of the circadian clock has also been implicated in the development and progression of metabolic-dysfunction-associated steatohepatitis (MASH)-related fibrosis, partly through dysregulation of TGF-β signaling [115]. Genetic variants in these pathways have been associated with T2D, obesity, hypertension, and MetS-related traits, supporting chronobiological mechanisms as an additional layer of genetic susceptibility in metabolic disorders [18,19] (Figure 1).

Overall, the genetic architecture of MetS reflects the contribution of multiple pathways involved in lipid metabolism, adipose tissue function, insulin signaling, and circadian regulation. Individual variants influence distinct components of this heterogeneous phenotype, highlighting the multifactorial nature of MetS and the complex interplay between genetic susceptibility and environmental factors in shaping cardiometabolic risk.

### 3.4. Genetic Determinants of Metabolic Dysfunction-Associated Steatotic Liver Disease

MASLD is characterized by hepatic steatosis, defined as fat accumulation in more than 5% of hepatocytes, in the presence of at least one cardiometabolic risk factor and absence of other specific causes of hepatic steatosis [116]. It encompasses a broad spectrum of disease severity, from simple steatosis to MASH, characterized by hepatic inflammation and hepatocellular injury and potentially progressing to liver fibrosis and, in advanced stages, cirrhosis [117].

Marked interindividual variability in hepatic fat accumulation and disease progression reflects the complex interplay between environmental factors and genetic susceptibility in MASLD [118]. Multiple genetic variants contribute to MASLD susceptibility through effects on hepatic lipid metabolism and transport, de novo lipogenesis, insulin resistance, and oxidative stress [118]. Among these, *PNPLA3* is one of the most extensively studied genes. It encodes adiponutrin, a protein involved in hepatic lipid remodeling and triglyceride metabolism [118]. The rs738409 C>G variant, resulting in an isoleucine-to-methionine substitution at position 148 (I148M), is the most extensively investigated *PNPLA3* variant and promotes hepatic lipid retention by altering protein activity [118,119]. It is also the most consistently replicated common genetic determinant of MASLD across diverse populations and contributes substantially to the heritable variability of hepatic steatosis [119]. A recent meta-analysis reported an approximately twofold higher risk of MASLD among GG genotype carriers, whereas the CC genotype was associated with an approximately 50% lower risk [120]. Another meta-analysis found that GG genotype carriers with MASLD had higher hazards of liver-related events and new-onset hepatocellular carcinoma (hazard ratio—HR = 2.29 and 2.25, respectively), with no significant associations with major cardiovascular events, extrahepatic cancers, or all-cause mortality [119].

Transmembrane 6 superfamily member 2 (*TM6SF2*), located on chromosome 19p12, encodes a transmembrane protein expressed in the liver, intestine, and kidney and involved in hepatic lipid and lipoprotein metabolism, although its precise function remains incompletely characterized [121]. The rs58542926 C>T (p.Glu167Lys, E167K) variant reduces VLDL-mediated lipid secretion, promoting hepatic fat accumulation while lowering circulating lipid levels [122]. A large meta-analysis reported that the rs58542926 T allele was associated with increased MASLD risk in both adults and children (OR = 1.62 and 2.87, respectively), with a substantially stronger effect in children [123]. The variant has also been associated with greater hepatic steatosis severity and fibrosis, as well as increased transaminase levels and reduced serum TG and cholesterol concentrations, reflecting its opposite effects on hepatic fat accumulation and circulating lipid levels [123,124].

The *GCKR* gene encodes glucokinase regulatory protein (GKRP), a key regulator of hepatic glucokinase (GCK) activity and glucose metabolism [125]. The rs1260326 C>T (p.Pro446Leu, P446L) variant alters GKRP function, enhancing hepatic glycolysis and increasing malonyl-CoA concentrations, thereby promoting de novo lipogenesis, inhibiting carnitine palmitoyltransferase 1 (CPT1), reducing fatty acid oxidation, and favoring triglyceride synthesis in hepatocytes [125]. Two common *GCKR* variants, rs780094 and the functional missense variant rs1260326, are in strong linkage disequilibrium (LD) and have been associated with fatty liver and other metabolic traits [125,126]. A recent meta-analysis confirmed an association between *GCKR* rs780094 and MASLD, with pooled ORs ranging from 1.18 to 1.38 across genetic models [126]. Notably, the rs1260326 TT genotype was associated with a markedly increased risk of progression to MASH among individuals with MASLD (OR = 16.8), together with elevated alanine aminotransferase levels and reduced markers of hepatic synthetic function, although these findings require confirmation in larger prospective cohorts [127] (Figure 1).

These findings underscore the contribution of genetic variation to MASLD susceptibility and progression. Variants affecting hepatic lipid handling, lipoprotein secretion, and glucose metabolism contribute to interindividual differences in hepatic fat accumulation and disease severity. Together, these observations highlight the multifactorial nature of MASLD and provide a biological basis for investigating gene–environment and gene–diet interactions in precision nutrition.

## 4. From Single Variants to Genetic Risk Scores

### 4.1. From GWAS Associations to Aggregate Genetic Risk

The emergence of precision medicine has stimulated increasing efforts to identify genetic and environmental factors influencing disease risk, with the aim of developing preventive strategies and tailored interventions for individuals at higher risk [31]. The widespread application of GWASs has led to the identification of more than 625,000 variant–trait associations across over 15,000 traits and disease outcomes [8]. These discoveries have expanded the catalogue of disease-associated variants, demonstrating that inherited susceptibility to complex diseases is shaped by hundreds or even thousands of common variants, each generally exerting a modest individual effect on disease risk [128]. GWAS-identified variants may help stratify individuals according to disease risk and potentially support earlier detection, preventive interventions, and personalized therapeutic approaches [129]. Studies across diverse ancestral populations have also revealed substantial heterogeneity in genetic susceptibility, which may contribute to differences in disease risk and clinical presentation [128,129].

However, GWAS-associated variants often explain only a limited proportion of the heritability of complex traits and do not necessarily correspond to the causal variants or genes underlying observed associations [129]. Multiple variants within the same genomic locus may also contribute to a given association, further complicating the identification of causal variants [128]. In addition, gene–gene and gene–environment interactions may contribute to some of the remaining unexplained genetic variation, although such interactions generally have modest effects and have proven difficult to replicate consistently [128,129]. These limitations highlight the complexity of the genetic architecture of complex traits and support the use of aggregate measures that capture the cumulative contribution of multiple genetic variants rather than their effects individually.

### 4.2. Genetic Scores: GRS, PGS, and PRS

The complexity of the genetic architecture of complex traits and the cumulative effects of multiple variants have prompted the development of approaches that combine genetic information into a single quantitative measure of susceptibility. These scores typically integrate multiple variants by weighting allele counts according to their effect sizes, generally derived from GWAS summary statistics [130,131]. They are broadly referred to as polygenic scores (PGSs), whereas the term polygenic risk score (PRS) is commonly used when assessing disease susceptibility or other clinically relevant outcomes [130]. However, terminology is not fully standardized, and terms such as genetic risk score and genomic risk score are also used for related approaches. In commonly used methods, variants are selected according to predefined *p*-value thresholds and combined to estimate genetic susceptibility [132]. Because nearby variants may be correlated through linkage disequilibrium (LD), PRS construction typically involves LD-based pruning or clumping to reduce redundancy and avoid overrepresentation of the same genetic signal [133].

By integrating the effects of multiple variants, PRSs can provide a more comprehensive measure of inherited predisposition than individual variants and may improve risk stratification for disease prevention and management [8]. Although population risk assessment should also incorporate lifestyle factors and biomarkers, PGSs can estimate genetic susceptibility from birth and may therefore contribute to early risk assessment [134]. Beyond disease-specific risk, genetic scores may also inform broader health outcomes, including life expectancy and disability-adjusted life-years [134]. At the individual level, the score provides a quantitative measure of relative lifetime genetic susceptibility, with higher scores indicating greater inherited predisposition to a given trait or disease [133]. When used for disease prediction, model discrimination can be assessed using the area under the receiver operating characteristic curve (AUC), which allows comparison of models with and without a genetic score [135,136]. The predictive accuracy of genetic scores depends on the heritability of the trait and how well the score captures its underlying genetic architecture, including the amount of genetic information and the variants incorporated into the model [136].

Despite their potential, polygenic scores face several challenges. A major concern is their transferability across ancestral populations, as many GWASs used to derive these scores have predominantly included individuals of European ancestry, resulting in reduced predictive performance in populations with different genetic backgrounds [133]. Conventional PRSs also primarily capture common GWAS-identified variants and may not adequately account for rare variants or gene–gene interactions, limiting their ability to represent the full genetic contribution to disease risk [137]. Moreover, although calculated at the individual level, PRSs primarily capture genetic susceptibility and do not directly incorporate environmental and lifestyle factors [137]. Their clinical utility therefore depends on whether they provide meaningful improvement beyond established clinical risk factors and remains under investigation [134]. Advances in machine learning and artificial intelligence may further enhance PRS-based models by integrating genetic, clinical, environmental, and lifestyle information and capturing complex interactions among these determinants [135].

### 4.3. Applications for Metabolic Diseases

**Figure 2:**
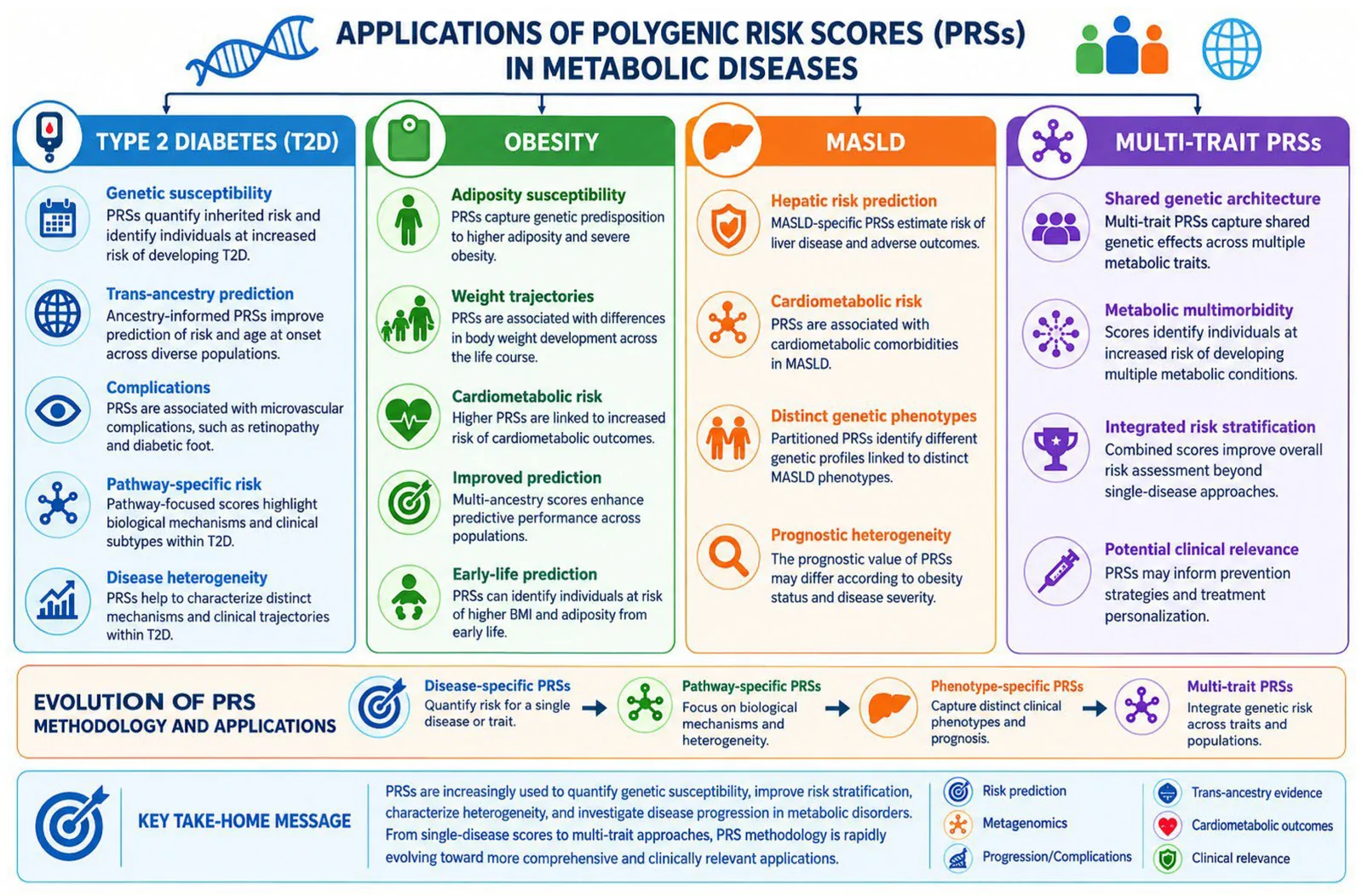
Applications of polygenic risk scores (PRSs) in metabolic diseases. Polygenic risk scores have been increasingly applied to quantify genetic susceptibility, improve risk stratification, characterize disease heterogeneity, and investigate disease progression across major metabolic disorders. Applications in type 2 diabetes (T2D), obesity, and metabolic dysfunction-associated steatotic liver disease (MASLD) have progressively expanded from disease-specific scores toward pathway-specific, phenotype-specific, and multi-trait approaches integrating shared genetic architecture across metabolic traits. Image created with the assistance of ChatGPT (OpenAI).

As discussed above, metabolic disorders are inherently multifactorial and involve complex biological mechanisms [138]. In this context, PRSs have emerged as useful tools for quantifying aggregate genetic risk, characterizing susceptibility to metabolic traits and disorders, and exploring the heterogeneity underlying these conditions [8,138]. Their application has been investigated particularly in major metabolic conditions, including T2D, obesity, and MASLD, although the extent and nature of the available evidence vary across diseases (Figure 2).

Among metabolic disorders, T2D represents one of the most extensively investigated applications of PRSs. A recent study reported that each standard deviation increase in the PRS was associated with a 1.37-year earlier age at T2D diagnosis and 43% higher odds of incident T2D at one-year follow-up [139]. These associations were also observed among Hispanic/Latino participants, supporting potential transferability across populations of different ancestries [139]. In the PREDIMED study, Corella et al. further showed that the association between the *TCF7L2* rs7903146 variant and T2D risk varied by obesity status, being stronger among non-obese than obese participants (HR = 1.81 vs. 1.01) [140]. To account for this heterogeneity, the authors developed obesity-specific genetic risk scores incorporating ten T2D-associated polymorphisms, which improved predictive performance within obesity strata compared with conventional approaches [140].

A trans-ancestry approach was also evaluated in a Taiwanese population, where a T2D PRS integrating GWAS data from East Asian and European populations explained 7.4% of the variation in T2D status and was inversely associated with age at disease onset, with a stronger association in males [141]. The PRS was also associated with selected diabetic complications, including retinopathy and diabetic foot, highlighting its potential to capture heterogeneity in disease progression [141]. Beyond overall susceptibility, pathway-specific PRSs have been used to characterize the genetic heterogeneity of T2D. In British Pakistani and British Bangladeshi individuals, partitioned polygenic scores for insulin deficiency and lipodystrophy were strongly associated with T2D and younger age at diagnosis [142]. Individuals with high genetic risk for both pathways were diagnosed with T2D 8.2 years earlier and at a 3 kg/m^2^ lower BMI than those with low genetic risk for both, and these scores were also associated with faster progression to insulin dependence and microvascular complications [142]. Similar pathway-specific approaches in other populations have further characterized distinct genetic components of T2D and their associations with disease progression and complications [143].

Beyond T2D, obesity represents another major area of PRS research. These scores have been used to quantify inherited susceptibility to adiposity and investigate associations with BMI, obesity risk, and weight trajectories across the life course [144]. Khera et al. developed a genome-wide polygenic score for BMI based on 2.1 million common variants, showing that higher scores were associated with substantial differences in body weight and increased risk of severe obesity [145]. In a longitudinal birth cohort, score-related differences in weight emerged during early childhood and increased progressively through adolescence, reaching approximately 12 kg by age 18 [145]. Higher scores were also associated with increased risks of T2D, hypertension, coronary heart disease, heart failure, and ischemic stroke, linking genetic susceptibility to adiposity with broader cardiometabolic risk [145]. More recently, Smit et al. developed a multi-ancestry PGS for BMI using genetic data from up to 5.1 million individuals. The score explained 17.6% of BMI variation among UK Biobank participants of European ancestry, more than twice the variance explained by the PGS developed by Khera et al. [145,146]. In children, higher PGSs were associated with accelerated BMI gain and earlier adiposity rebound, and adding the PGS to early-life measures improved prediction of BMI at age 18, supporting its potential utility for early identification of individuals at higher risk of obesity [146].

In MASLD, polygenic scores have been used not only to assess genetic susceptibility to hepatic fat accumulation but also to investigate disease heterogeneity and progression. Xiao et al. developed a MASLD-specific PRS and showed that higher genetic risk was associated with severe liver disease and cardiometabolic outcomes among individuals with MASLD, including severe liver disease (HR = 3.15), T2D (HR = 2.81), and coronary artery disease (HR = 1.36) [147]. These findings suggest that polygenic risk may provide additional prognostic information beyond hepatic steatosis alone. Jamialahmadi et al. subsequently developed two partitioned PRSs based on distinct genetic associations with intrahepatic lipoprotein retention, identifying at least two genetic profiles of MASLD: one associated with more aggressive liver disease but limited cardiometabolic burden, and another characterized by a less severe hepatic phenotype but greater cardiometabolic susceptibility [148]. Similarly, a recent multicentre Japanese cohort study found that the prognostic relevance of a two-variant PRS differed according to obesity status. Although the PRS independently predicted liver-related events in both obese and non-obese individuals with MASLD, its association with overall survival was observed only among non-obese patients [149]. Collectively, these findings suggest that polygenic risk in MASLD may extend beyond disease susceptibility and help characterize heterogeneous disease phenotypes and prognostic profiles.

A further evolution of PRS methodology was proposed by Kim et al., who developed composite scores integrating multi-ancestry GWAS data across 20 metabolic traits from more than 8.5 million individuals [138]. The resulting O-MetPRS and D-MetPRS, optimized for obesity and T2D, respectively, outperformed existing disease-specific PRSs across six ancestries and identified individuals at increased risk of metabolic multimorbidity [138]. Notably, individuals in the highest decile of either score had approximately twice the risk of initiating glucagon-like peptide-1 receptor agonist treatment compared with those in the middle quintile [138]. By capturing the shared genetic architecture of multiple metabolic traits, these integrated scores may provide a broader assessment of inherited metabolic susceptibility than disease-specific PRSs (Table 1).

## 5. Mediterranean Diet–Gene Interactions in Metabolic Diseases

The MedDiet originally referred to the dietary patterns traditionally followed in olive-growing areas of the Mediterranean region and is now broadly recognized as a dietary pattern encompassing regional variations across the Mediterranean basin [150]. Despite these regional variations, the MedDiet is generally characterized by a high consumption of plant-based foods, olive oil, whole grains, legumes, nuts, and fish, together with moderate consumption of dairy products and poultry and a low intake of red and processed meat [150]. From a macronutrient perspective, the MedDiet is generally characterized by a relatively high proportion of fat, predominantly provided by monounsaturated fatty acids (MUFA), with a lower contribution from saturated fatty acids (SFA). For example, in the CORDIOPREV trial, fat accounted for 35% of total energy intake in the MedDiet, including 22% from MUFA, 6% from polyunsaturated fatty acids (PUFA), and 7% from SFA [151]. Evidence from long-term observational studies and randomized controlled trials (RCTs) supports the beneficial effects of the MedDiet on cardiometabolic and liver health, including improvements in lipid profile and adiposity, reduced incidence of T2D and MetS, and beneficial effects on hepatic steatosis and liver-related metabolic outcomes [152,153]. Within the framework of nutrigenetics, which seeks to understand how genetic variation contributes to inter-individual differences in response to dietary factors, the effects of the MedDiet may vary according to an individual’s genetic background, supporting a potential role for gene–diet interactions in modulating cardiometabolic risk [150,154]. However, it should be noted that the MedDiet does not represent a single standardized dietary exposure. Across studies, adherence has been assessed using different dietary indices and scoring systems, while intervention studies have employed diverse protocols ranging from Mediterranean dietary patterns alone to diets supplemented with extra-virgin olive oil (EVOO) or nuts. Consequently, the term MedDiet may encompass heterogeneous dietary exposures, which should be considered when comparing findings across studies and interpreting gene–diet interactions. Furthermore, EVOO, one of the hallmark components of the MedDiet, may contribute to its beneficial metabolic effects through both its high monounsaturated fatty acid content and the presence of bioactive compounds such as polyphenols [154].

This has prompted growing interest in whether adherence to the MedDiet can modify the phenotypic expression of genetic susceptibility to metabolic traits and diseases. Early studies primarily investigated interactions between the MedDiet and individual genetic variants, whereas subsequent approaches have progressively extended this framework to genome-wide analyses and aggregate measures of genetic susceptibility, such as GRSs. It should be noted that the studies included in this review assessed heterogeneous dietary exposures. While some investigations evaluated adherence to the Mediterranean dietary pattern as a whole through MedDiet scores or dietary interventions, others focused on specific dietary components commonly associated with the MedDiet, including olive oil, MUFA intake, nuts, or dietary fat quality. Therefore, gene–nutrient interactions involving these individual components should not be considered equivalent to gene–MedDiet interactions, and findings should be interpreted in light of the specific dietary exposure assessed in each study.

Importantly, associations between genetic susceptibility and metabolic outcomes, as well as associations between adherence to the Mediterranean dietary pattern and metabolic outcomes, should be distinguished from formal gene–diet interactions. Throughout this review, studies reporting statistically significant interaction terms are described as evidence of gene–diet interactions, whereas studies reporting only independent genetic or dietary associations, genotype-specific associations, or differential responses without a statistically significant interaction are discussed accordingly.

An additional consideration when interpreting the evidence is the ancestry and population background of the study participants. A substantial proportion of the available literature, particularly candidate-gene studies and Mediterranean dietary intervention studies, derives from populations of predominantly European ancestry. Therefore, findings observed in these populations may not be directly generalizable to individuals from other ancestral backgrounds, and the magnitude or direction of gene–diet associations may differ across populations. This issue is particularly relevant to GRSs and PRSs, whose performance and transferability may vary across ancestries depending on the populations in which the underlying genetic associations were identified and the scores were developed.

### 5.1. Evidence from Candidate-Gene Studies

#### 5.1.1. Type 2 Diabetes

Candidate-gene studies provided some of the earliest evidence of gene–MedDiet interactions. In a large case–control study of 7052 participants at high cardiovascular risk, Ortega-Azorín et al. reported significant interactions between *FTO* rs9939609 and *MC4R* rs17782313 and MedDiet adherence in relation to T2D risk [155]. Among participants with a low adherence to the MedDiet, the risk variants were associated with a higher T2D risk for both *FTO* and *MC4R*, whereas these associations were substantially attenuated among those with high adherence; significant interactions were observed for *FTO*, *MC4R*, and their aggregate genetic score [155]. The authors also investigated whether folate intake contributed to the observed gene–diet interaction. Although no significant interaction between folate intake and either polymorphism was observed for T2D, *FTO* rs9939609 significantly interacted with folate intake in determining fasting glucose concentrations among non-diabetic participants, with attenuation of the genetic effect at higher folate intake [155]. However, these findings were considered preliminary, as folate intake may partly reflect overall dietary quality, and its independent contribution remains uncertain.

Within PREDIMED, a multicenter clinical trial, Corella et al. reported that prerandomization adherence to the MedDiet modified the association between the *TCF7L2* rs7903146 polymorphism and fasting glucose concentrations, even after adjustment for T2D, BMI, and other confounders [156]. The *TCF7L2* rs7903146 polymorphism was significantly associated with T2D, while TT carriers exhibited higher fasting glucose concentrations than CC + CT carriers only among participants with low adherence to the MedDiet; this difference was no longer observed among those with high adherence [156]. Similar interaction patterns were also observed for TG, total cholesterol, and low-density lipoprotein (LDL) cholesterol, suggesting that greater adherence to the MedDiet may attenuate the adverse metabolic profile associated with the risk genotype [156].

More recently, a short-term randomized crossover intervention trial investigated whether *TCF7L2* rs7903146 influenced lipoprotein responses to a MedDiet compared with a low-fat diet [30]. Differences in VLDL particle profiles according to *TCF7L2* genotype were observed following the low-fat diet but not after the MedDiet [30]. However, the formal *TCF7L2* × diet interaction did not reach statistical significance. Thus, these findings suggest genotype-dependent differences in response to the dietary interventions but do not provide statistically significant evidence of a gene–diet interaction [30]. Earlier crossover feeding studies also reported genotype-dependent differences in insulin sensitivity following Mediterranean-type, olive oil-rich dietary interventions. Variants in *SCARB1*, encoding scavenger receptor class B type I, a receptor involved in the hepatic uptake and metabolism of lipoproteins, and *LIPC*, encoding hepatic lipase, an enzyme involved in the metabolism of circulating lipoproteins, were associated with genotype-dependent differences in insulin sensitivity following MUFA-rich dietary interventions [157,158]. These findings provide supportive evidence of genotype-dependent dietary responses, although they should be distinguished from studies demonstrating a statistically significant gene–diet interaction and interpreted cautiously given the limited evidence base.

Beyond genes primarily involved in glucose and energy metabolism, variation in circadian clock genes has also been investigated in relation to metabolic health, reflecting the role of circadian regulation in glucose and lipid homeostasis [159]. In a large longitudinal analysis within the PREDIMED randomized trial, Corella et al. found that carriers of the *CLOCK* rs4580704 G allele had a lower risk of developing T2D than CC homozygotes [17]. This protective effect was more pronounced among participants assigned to the MedDiet intervention than in the control group, although the gene–diet interaction was only borderline significant [17]. The borderline interaction therefore suggests a potential effect modification rather than providing conclusive evidence of a gene–diet effect. This observation is consistent with previous evidence linking *CLOCK* rs4580704 variation to glucose homeostasis and insulin sensitivity in response to dietary fatty acid composition [160].

Overall, the available evidence suggests that genetic variation may modify metabolic responses to the MedDiet and its major components, particularly in relation to T2D and related metabolic traits. However, the strength of the evidence varies across studies depending on the statistical evidence for interaction. Some studies have demonstrated statistically significant gene–diet interactions, whereas others have reported genotype-dependent responses without a significant interaction term or only borderline evidence of interaction. Collectively, these observations support a potential role of genetic variation in modifying responses to the MedDiet, but their reproducibility and clinical relevance remain to be established (Table 2).

#### 5.1.2. Obesity and Adiposity

In one of the first longitudinal studies investigating whether a Mediterranean-style diet could modify the association between the *FTO* rs9939609 polymorphism and body weight, Razquin and colleagues observed that subjects with the AA genotype had the highest body weight at baseline [29]. Nevertheless, after the 3-year nutritional intervention, carriers of the A allele showed lower body weight gain than wild-type TT subjects [29]. The association between the A allele and lower body weight gain was statistically significant among participants assigned to the MedDiet groups supplemented with virgin olive oil or nuts, but not among those assigned to the conventional low-fat control diet [29]. However, the formal gene–diet interaction test was not statistically significant, indicating that these findings should be interpreted cautiously [29]. Further evidence for the potential modulation of genetic susceptibility to obesity by lifestyle factors was provided by Corella et al. in the PREDIMED study involving 7052 participants [28]. The *FTO* rs9939609 and *MC4R* rs17782313 variants were jointly evaluated in relation to obesity and their potential interaction with diet and physical activity [28]. Each additional risk allele in the aggregate score was associated with a 7% increase in the odds of obesity, whereas greater adherence to the MedDiet appeared to attenuate this association, although no statistically significant gene–diet interaction was observed [28]. Notably, among individuals carrying three or four risk alleles, higher adherence to the MedDiet was associated with BMI values comparable to those observed in individuals without risk alleles and lower MedDiet adherence [28]. Despite the observed attenuation of obesity-related genetic susceptibility with higher MedDiet adherence, the formal gene–diet interaction was not statistically significant [28]. Therefore, these findings should be interpreted as evidence of independent genetic and dietary associations rather than as confirmation of a true gene–diet interaction [28]. In a randomized clinical trial involving 188 Italian subjects, Di Renzo et al. investigated whether *FTO* rs9939609 modified the response to a 4-week MedDiet intervention [161]. A significant gene–diet interaction was observed for the change in total body fat, with A-allele carriers showing a greater reduction in total body fat than TT carriers within the MedDiet group [161]. The MedDiet was also associated with a greater reduction in gynoid body fat regardless of *FTO* rs9939609 genotype, with no significant gene–diet interactions [161]. Although these findings provide evidence of a genotype-dependent response, their interpretation is limited by the relatively small sample size.

A large case–cohort study involving 11,048 participants from five European countries investigated whether *FTO* rs9939609 and *TCF7L2* rs7903146 modified the associations between the Mediterranean diet score (MDS) and Nordic diet score and changes in obesity-related traits [162]. Specifically, by comparing individuals with the greatest degree of unexplained weight gain during a median follow-up of 6.8 years with control subjects, higher MDS was associated with a decrease in WC and WC adjusted for BMI per 1-point increment but not with weight change [162]. While *FTO* did not modify these associations, a borderline interaction between MDS and *TCF7L2* rs7903146 was observed for weight gain, with a beneficial association of greater MedDiet adherence among carriers of one or two risk alleles [162]. The observed pattern suggests a possible genotype-specific association between MedDiet adherence and obesity-related outcomes beyond *FTO*, although evidence for a true gene–diet interaction involving *TCF7L2* remains limited and warrants replication. Conversely, a cross-sectional analysis involving 1120 Puerto Rican adults reported significant interactions between *TCF7L2* rs7903146 and rs12255372 risk variants and MedDiet adherence in relation to anthropometric measures [163]. Carriers of the rs7903146 T risk allele had lower weight and WC with high MedDiet adherence, although a borderline interaction was observed for BMI [163]. Similarly, a significant interaction between *TCF7L2* rs12255372 and MedDiet adherence was observed for BMI and weight, but not for WC [163]. The observed associations were further supported among carriers of risk alleles at both *TCF7L2* loci, who exhibited significantly lower anthropometric measures at high MedDiet adherence than carriers of the common GC haplotype, supporting a potential modifying effect of MedDiet adherence on the phenotypic expression of *TCF7L2*-related genetic susceptibility [163]. However, because these data were derived from a cross-sectional analysis, they require confirmation in prospective and intervention studies.

Finally, a cross-sectional study of 392 adults living in the Mediterranean basin examined whether adherence to the MedDiet modified the associations of *FTO* rs9939609, *MC4R* rs17782313, and *MTHFR* rs1801133 (C677T) with obesity [164]. *MTHFR* encodes methylenetetrahydrofolate reductase, an enzyme involved in folate metabolism, and the C677T variant is associated with reduced enzymatic activity and has been investigated as a potential genetic determinant of obesity susceptibility [165]. Aoun et al. found that *FTO* rs9939609 was significantly associated with waist-to-hip ratio (WHR), whereas *MC4R* rs17782313 was associated with obesity-related anthropometric traits [164]. No consistent associations were detected for *MTHFR* rs1801133 [161]. Importantly, a greater adherence to the MedDiet did not appear to confer protection against overweight or obesity among genetically predisposed individuals [164].

In another 3-year intervention study, Razquin et al. investigated whether the interleukin-6 gene (*IL6*) −174G/C (rs1800795) polymorphism, which has been associated with circulating IL-6 levels and cardiometabolic risk [166,167], modified changes in body weight in response to a MedDiet enriched with virgin olive oil among individuals at high cardiovascular risk [168]. At baseline, participants with the CC genotype had greater adiposity measures [168]. After 3 years, however, CC carriers assigned to the MedDiet enriched with virgin olive oil showed the lowest weight gain, with a significant interaction between the dietary intervention and genotype [168].

Collectively, studies of candidate genes suggest that adherence to the MedDiet may influence the phenotypic expression of inherited susceptibility to obesity. Statistically significant gene–diet interactions have been reported for selected variants, including *FTO*, *TCF7L2*, and *IL6*, although results have not been consistent across studies. Several other studies have reported genotype-specific associations or differential responses to dietary interventions without formal evidence of interaction, while some have found no clear modification of genetic susceptibility by MedDiet adherence. Overall, the available evidence should be interpreted in light of both the statistical evidence for interaction and the underlying study design, while the clinical relevance and reproducibility of effects based on candidate genes remain uncertain (Table 3).

#### 5.1.3. Metabolic Syndrome

Evidence of gene–diet interactions has also been investigated in relation to MetS. Although several studies have explored this topic, findings remain heterogeneous owing to differences in the genetic variants examined, dietary exposures considered, and MetS-related outcomes assessed. Among candidate-gene studies relevant to MetS, Ortega-Azorín et al. investigated whether variation in the Max-like protein X interacting protein-like (*MLXIPL*) gene modified the association between MedDiet adherence and TG levels and hypertriglyceridemia in participants of the PREDIMED trial [169]. *MLXIPL* encodes carbohydrate-responsive element-binding protein (ChREBP), a transcription factor involved in carbohydrate and lipid metabolism, and the rs3812316 polymorphism has been associated with lower TG levels [170,171]. Accordingly, G-allele carriers had significantly lower baseline TG levels and were 37% less likely to present with hypertriglyceridemia than CC homozygotes, particularly among participants with high MedDiet adherence [169]. During follow-up, both the *MLXIPL* rs3812316 polymorphism and the MedDiet were independently associated with lower TG concentrations [169]. A significant interaction between rs3812316 and baseline MedDiet adherence was observed for hypertriglyceridemia, whereas no statistically significant effect modification by the dietary intervention itself was detected during follow-up [169]. The authors also reported evidence consistent with a biological interaction, with G-allele carriers assigned to the MedDiet exhibiting lower TG concentrations and a lower incidence of myocardial infarction than G-allele carriers in the control group; however, this observation was not supported by statistically significant effect modification by the dietary intervention during follow-up [169].

The interaction between genetic variation in inflammatory pathways and MedDiet adherence was investigated in relation to MetS in the CORDIOPREV study, a prospective randomized trial in patients undergoing secondary cardiovascular prevention [172]. In this context, two candidate variants (rs1800629 and rs1799964) in the tumor necrosis factor alpha (*TNF-α*) gene, encoding a proinflammatory cytokine involved in the regulation of a wide range of biological processes and diseases [173], were examined for their potential role in modifying the association between MedDiet adherence and MetS and its metabolic components, although the main finding was observed for rs1800629 [172]. At baseline, individuals with the GG genotype at rs1800629 had significantly higher fasting and postprandial TG and high-sensitivity C-reactive protein (hsCRP) concentrations than A-allele carriers (GA + AA) [172]. After 12 months of MedDiet, these baseline differences between genotypes disappeared, while the decreases in TG and hsCRP concentrations were significantly greater in GG subjects than in A-allele carriers [172]. These findings indicate genotype-dependent responses to the MedDiet, although they should not be interpreted as evidence of a statistically significant gene–diet interaction in the absence of a reported interaction test.

The role of genetic variation in lipid metabolism was also explored in the context of different dietary patterns, with particular attention to the cholesteryl ester transfer protein gene (*CETP*) rs3764261 polymorphism [174], which has been associated with higher HDL cholesterol levels [175,176]. Garcia-Rios et al. reported a significant gene–diet interaction between the rs3764261 polymorphism and dietary pattern for both HDL cholesterol and TG concentrations after 1 year of intervention [174]. In particular, carriers of the minor T allele displayed significantly higher plasma HDL cholesterol and lower TG levels than GG homozygotes following the MedDiet, whereas no significant genotype-related differences were observed following the low-fat diet [174].

Beyond lipid metabolism-related genes, gene–diet interactions have also been investigated for genes involved in adipose tissue function and metabolic regulation. Coltell et al. investigated whether selected *ADIPOQ* variants modified the association between MedDiet adherence and circulating adiponectin levels in high-cardiovascular-risk participants of the PREDIMED trial [177]. In the whole population, adiponectin was positively associated with HDL cholesterol and inversely correlated with TG and fasting glucose, while higher circulating levels were associated with a 6.1-fold lower risk of developing T2D [177]. Among the four pre-selected *ADIPOQ* candidate SNPs, the promoter variant −11391G>A (rs17300539) showed the strongest association with plasma adiponectin concentrations, with the minor allele associated with significantly higher levels in the multivariable-adjusted model [177]. However, no significant interactions between these variants and MedDiet adherence were observed, and plasma adiponectin levels did not differ significantly between participants with low and high adherence levels [177]. Complementary evidence has also emerged from interventions targeting dietary fat quality. In the RISCK study, AlSaleh et al. reported a significant interaction between *ADIPOQ* variants and dietary fat composition on adiponectin concentrations, with the high-MUFA diet producing a more favorable response in specific genotype groups among individuals at risk of MetS [178]. Similarly, de Luis et al. reported genotype-dependent metabolic responses to hypocaloric diets differing in fatty acid composition [179]. Although both dietary interventions improved anthropometric parameters, only GG homozygotes showed significant increases in adiponectin concentrations together with greater improvements in insulin and Homeostaticsis Model Assessment for Insulin Resistance (HOMA-IR) after both the MUFA-rich and PUFA-rich diets, supporting genotype-dependent differences in metabolic responses to dietary fat quality [179]. Overall, these studies provide complementary evidence of genotype-dependent differences in response to dietary fat quality, while remaining distinct from the statistically significant gene–diet interactions reported in studies evaluating the broader Mediterranean dietary pattern.

An Iranian case–control study evaluated the vascular endothelial growth factor (*VEGF*) +405 G/C (rs2010963) polymorphism and reported differences in Mediterranean Dietary Quality Index components according to genotype, with MetS patients carrying the CC genotype showing a higher saturated fatty acid score than carriers of the other genotypes [180]. This association is consistent with previous evidence linking the *VEGF* +405 G/C polymorphism to coronary artery disease in patients with type 2 diabetes [181]. However, the absence of a formal gene–diet interaction analysis limits the interpretation of these findings in the context of nutrigenetic modulation of MetS risk [180].

Further evidence of gene–diet interactions in relation to MetS has emerged from studies of circadian clock genes. Garaulet et al. reported significant interactions between *CLOCK* polymorphisms and dietary fatty acid composition, particularly MUFA intake, on glucose metabolism and insulin resistance [160]. For the rs4580704 polymorphism, the protective effect of the minor allele on insulin sensitivity was observed only among individuals with higher MUFA intake (>13.2% of energy), suggesting that dietary fat quality may modulate the metabolic effects of *CLOCK* variation [160]. Building on previous evidence linking *CLOCK* variation to the metabolic response to MUFA intake, Garcia-Rios et al. investigated whether three *CLOCK* polymorphisms (rs1801260, rs3749474, and rs4580704) interacted with dietary patterns to influence glucose and lipid metabolism among patients with MetS participating in the CORDIOPREV trial [151]. After 1 year of dietary intervention, a significant gene–diet interaction between rs1801260 and dietary pattern was observed for insulin concentration, HOMA-IR, and the quantitative insulin sensitivity check index (QUICKI) [151]. In the MedDiet group, TT homozygotes tended to have higher plasma insulin concentrations and HOMA-IR and lower insulin sensitivity than carriers of the minor C allele, although these differences were not statistically significant [151]. In contrast, after 1 year of the low-fat intervention, TT homozygotes exhibited a more favorable insulin profile than C-allele carriers, with lower plasma insulin concentrations and HOMA-IR and higher insulin sensitivity, consistent with the findings of Garaulet et al. [151,160].

Taken together, the available evidence suggests that genetic variation may influence metabolic responses to dietary patterns and specific dietary components in MetS. However, statistically significant gene–diet interactions have been demonstrated only for a subset of genetic variants and metabolic outcomes. Other studies have reported genotype-specific associations or differential responses to dietary interventions without demonstrating a statistically significant interaction, while some have found no evidence of effect modification by MedDiet adherence. Overall, these findings should be interpreted in light of both the statistical evidence for interaction and the underlying study design, with careful distinction between genotype-dependent responses and formally demonstrated gene–diet interactions (Table 4).

#### 5.1.4. Metabolic Dysfunction-Associated Steatotic Liver Disease

Given the strong genetic contribution to MASLD susceptibility, increasing attention has been devoted to whether dietary patterns such as the MedDiet may modulate the phenotypic expression of genetic risk. In particular, gene–diet interactions involving variants in key genes related to hepatic lipid metabolism have been examined in relation to liver fat accumulation and MASLD-related traits. Chen et al. investigated whether genetic variation in *PNPLA3* and *TM6SF2* modified the association between adherence to the MedDiet and liver fat content (LFC) in 21,619 UK Biobank participants [182]. Greater adherence to a Mediterranean-style diet was associated with lower LFC, while the *PNPLA3* G and *TM6SF2* T alleles were associated with higher LFC [182]. Significant interactions between MedDiet adherence and both *PNPLA3* and *TM6SF2* were observed, indicating that the association between MedDiet adherence and LFC varied according to genotype [182]. For *PNPLA3*, the association between MedDiet adherence and LFC was up to 3.8-fold greater in GG homozygotes than in CC homozygotes, providing evidence that the magnitude of the association varied according to genetic background [182]. More recently, Troisi et al. evaluated the effects of a personalized hypocaloric MedDiet in a prospective cohort of genetically characterized patients with MASLD, including carriers of variants in *PNPLA3*, *TM6SF2*, *MBOAT7* (membrane-bound O-acyltransferase domain-containing 7), and *GCKR* [183]. After six months, significant reductions in body weight, hepatic steatosis, and several metabolic parameters were observed [183]. Notably, *PNPLA3* risk-allele carriers experienced greater reductions in hepatic steatosis than non-carriers [183]. However, these genotype-specific differences were derived from genotype-stratified analyses, and no formal gene–diet interaction analysis was reported [183].

A Turkish single-center randomized controlled study of 250 overweight or obese adults with MASLD compared a 12-week moderately hypocaloric MedDiet with a low-fat diet [184]. Both diets significantly reduced controlled attenuation parameter (CAP), a measure of hepatic steatosis, liver stiffness measurement (LSM), a surrogate marker of liver fibrosis, and body weight after the intervention, with no significant differences between the two dietary groups [184]. However, no significant interactions between *PNPLA3* genotype and dietary intervention were observed for CAP, LSM, or body weight, indicating that the hepatic and weight-related responses to the two diets were not significantly influenced by *PNPLA3* genotype [184].

Taken together, evidence for gene–diet interactions in MASLD remains limited. Studies to date suggest that genetic background, particularly variants involved in hepatic lipid metabolism such as *PNPLA3*, may be associated with differential responses to Mediterranean dietary interventions, but formal evidence of gene–diet interaction remains scarce. Current evidence is therefore insufficient to support candidate gene-based dietary stratification in clinical practice. Further prospective studies and randomized dietary intervention studies, preferably in diverse populations and with adequate statistical power, are needed to clarify whether and to what extent genetic variation modifies the metabolic and hepatic response to the MedDiet in MASLD (Table 5).

### 5.2. Evidence from GRS Studies

The investigation of gene–diet interactions has also expanded from individual genetic variants to aggregate measures of genetic susceptibility. By combining the effects of multiple variants, these approaches may provide a more comprehensive assessment of genetic susceptibility and allow investigation of whether adherence to the MedDiet modifies the phenotypic expression of an overall genetic predisposition. Compared with candidate-gene approaches, genetic score studies may provide a broader representation of the polygenic architecture of complex metabolic traits and diseases. However, the available evidence remains relatively limited and heterogeneous, with differences in score construction, genetic variants included, dietary assessment, ancestry and population characteristics, and metabolic outcomes.

In T2D, one of the earliest studies to evaluate whether overall genetic susceptibility could be modified by adherence to the MedDiet was conducted within the EPIC-InterAct study [185]. Langenberg et al. developed a GRS based on 49 T2D-associated variants and examined its association with incident T2D in relation to adherence to a Mediterranean dietary pattern [185]. The GRS was strongly associated with incident T2D, confirming the contribution of cumulative genetic susceptibility to disease risk [185]. However, no significant GRS × MedDiet interaction was observed [185], suggesting that the association between the GRS and T2D incidence was largely independent of MedDiet adherence [185]. In a more recent longitudinal analysis including European American and African American adults, Hardy et al. investigated the interplay between ancestry-specific T2D PRSs, dietary patterns, and incident T2D [186]. Adherence to Mediterranean and Dietary Approaches to Stop High Blood Pressure (DASH) dietary patterns was associated with a lower risk of incident T2D, whereas the Southern dietary pattern was associated with increased risk [186]. High T2D PRSs were independently associated with increased T2D risk [186]. Although Mediterranean and DASH dietary patterns did not completely offset the excess risk associated with a high-risk PRS, predicted probability analyses showed the lowest probabilities of incident T2D among individuals with a high metabolic burden in the highest MedDiet tertile and the second DASH tertile across PRS strata [186]. However, no formal gene–diet interaction estimate was reported.

In obesity, aggregate measures of genetic susceptibility have also been investigated in relation to adherence to the MedDiet. Hosseini-Esfahani et al. conducted a nested case–control study within the Tehran Lipid and Glucose Study and generated a weighted *FTO*-based GRS including six obesity-associated variants [187]. A significant GRS × MedDiet interaction was observed for obesity risk, with higher adherence to the MedDiet associated with a progressive reduction in obesity risk among participants with higher genetic susceptibility (GRS ≥ 6), whereas no such trend was observed among those with lower genetic risk, suggesting a potential attenuation of *FTO*-related obesity susceptibility by adherence to the MedDiet [187]. Conversely, no significant interactions were detected for abdominal obesity or WHR [187]. A Swiss population-based cohort study evaluated obesity GRSs based on 31 and 68 obesity-associated variants in relation to dietary patterns, including two MedDiet scores [188]. While healthier dietary patterns and greater adherence to the MedDiet were associated with several favorable obesity-related outcomes, no significant interactions were observed between obesity GRSs and MedDiet scores, indicating that the dietary effects were largely independent of genetic susceptibility [188]. In European adolescents from the HELENA study, Seral-Cortes et al. examined the interaction between MedDiet adherence and an obesity GRS comprising 21 obesity-associated SNPs [32]. Significant obesity GRS × MedDiet interactions were observed for BMI, WC, and the MetS score, with stronger interaction effects generally observed in females than in males [32]. The same authors subsequently explored whether changes in MedDiet adherence and its individual components could modify genetic susceptibility to obesity, assessed using a BMI-PRS derived from more than two million variants in individuals of European ancestry, in a European pediatric population followed in both cross-sectional and longitudinal analyses [33]. While at baseline, MedDiet adherence appeared to exacerbate the association between BMI-PRS and BMI, six years later, during early adolescence, it showed an attenuating effect [33]. These contrasting patterns suggest that the association between dietary adherence and genetically predicted obesity risk may vary across developmental stages, although they should not be interpreted as evidence of a formal gene–diet interaction in the absence of a significant interaction test.

Evidence from GRS-based studies in metabolic syndrome remains scarce. In the Food4Me study, San-Cristóbal et al. evaluated the interaction between adherence to the MedDiet and a multi-trait GRS comprising 14 variants associated with metabolic syndrome-related traits [186]. Greater baseline adherence to the MedDiet was associated with larger improvements in BMI, WC, and glucose concentrations over the 6-month intervention [189]. However, evidence for a gene–diet interaction was limited, with a significant GRS × MedDiet interaction observed only for circulating carotenoid concentrations, whereas no significant interactions were detected for the main MetS components [189]. These findings suggest that the beneficial effects of the MedDiet on metabolic traits were largely independent of the underlying genetic risk score.

Beyond the candidate-gene analyses, Chen et al. also examined a hepatic steatosis PRS in a large UK Biobank cohort [182]. Extending their candidate-gene analyses, greater adherence to a Mediterranean dietary pattern was associated with lower LFC, whereas higher genetic susceptibility was associated with increased steatosis [182]. Importantly, the authors observed significant interactions between the PRS and MedDiet adherence, indicating that the inverse association between the Mediterranean dietary pattern and LFC varied according to genetic susceptibility [182]. Similar interaction patterns were also observed for cT1, a marker of liver inflammation and fibrosis, suggesting that PRS-based approaches may help identify differences in hepatic responses to the MedDiet in MASLD [182]. In contrast, Troisi et al. evaluated a weighted polygenic risk score including *PNPLA3*, *TM6SF2*, *MBOAT7*, and *GCKR* variants in patients with MASLD undergoing a personalized hypocaloric MedDiet intervention [183]. Although significant improvements in hepatic steatosis and metabolic parameters were observed following weight loss, no significant association was detected between the composite PRS and steatosis reduction [183]. These findings suggest that the predictive value of aggregate risk scores for dietary response in MASLD remains uncertain.

Overall, evidence on interactions between MedDiet adherence and genetic risk scores across metabolic diseases remains limited and heterogeneous. Across obesity, metabolic syndrome, and T2D, greater adherence to Mediterranean dietary patterns is generally associated with more favorable metabolic outcomes, whereas statistically significant gene–diet interactions have been demonstrated only in a subset of studies and outcomes. Several studies have instead reported independent genetic and dietary associations without formal evidence of interaction. In MASLD, evidence from a large population-based study indicates that the association between MedDiet adherence and lower liver fat varied according to genetic susceptibility. However, this evidence currently relies on a limited number of studies and has not been consistently replicated. Therefore, although GRS- and PRS-based approaches may contribute to the development of precision nutrition strategies, the current evidence remains insufficient to support genetically guided dietary recommendations in clinical practice. Further prospective cohort studies and randomized dietary intervention studies, preferably in diverse populations and with adequate statistical power, are needed to determine whether genetic risk scores can reliably identify individuals who derive differential metabolic or hepatic benefits from the MedDiet (Table 6).

### 5.3. Cross-Disease Synthesis

Taken together, the evidence across T2D, obesity, MetS, and MASLD indicates that the potential for genetic background to modify the metabolic effects of the MedDiet is biologically plausible but remains inconsistently demonstrated. Candidate-gene studies currently account for the larger body of evidence, with statistically significant interactions reported across T2D, obesity, and MetS, although these findings are heterogeneous and often lack independent replication. GRS- and PRS-based approaches provide a broader assessment of genetic susceptibility but remain less extensively studied. The most consistent evidence for effect modification comes from obesity and MASLD, although even in these conditions, significant interactions have been reported only in a subset of studies. In contrast, evidence for T2D and MetS is more heterogeneous, with several studies reporting independent genetic and dietary associations or genotype-dependent responses without formal evidence of interaction. Overall, the available literature supports a potential contribution of genetic susceptibility to interindividual variation in response to the MedDiet, but does not yet establish reproducible or clinically actionable gene–diet interactions across metabolic diseases (Table 7).

## 6. Challenges and Future Perspectives

A major finding emerging from this review is the substantial heterogeneity of the available evidence, which complicates direct comparisons across studies and limits the possibility of drawing definitive conclusions. Even within the same metabolic disease, studies differed considerably in design, population characteristics, sample size, duration of follow-up, dietary assessment methods, and outcome definitions. These differences also affect the strength and generalizability of the evidence, particularly when considering statistical power, population ancestry, replication across independent cohorts, and whether a formal gene–diet interaction was demonstrated. Furthermore, genetic susceptibility was evaluated using different approaches, ranging from candidate-gene analyses to GRSs and PRSs, often constructed using different variants, weighting strategies, and risk models. As a result, findings are difficult to compare directly, and their consistency remains challenging to evaluate.

An additional source of variability lies in the diversity of disease-related outcomes evaluated across studies. Even within the same metabolic condition, investigations have focused on different phenotypic traits and biomarkers, including anthropometric measures in obesity, glycemic and disease-related endpoints in T2D, and imaging-, biochemical-, or metabolomics-based markers in MASLD [30,32,156,182,183,184]. This heterogeneity may contribute to the inconsistent findings reported for gene–diet interactions.

Candidate-gene and GRS/PRS approaches also provide partly different perspectives. Candidate-gene studies may offer mechanistic insights into specific biological pathways but are inherently limited by their focus on a small number of variants. Although candidate-gene studies have provided important initial insights into potential biological mechanisms underlying gene–diet interactions, their findings should be interpreted with caution. Many candidate-gene associations have not been consistently replicated across independent populations, and several studies were conducted in relatively small samples, increasing the risk of false-positive findings and limiting generalizability. Consequently, the strength and generalizability of evidence from candidate-gene studies may be limited compared with approaches based on GRSs and PRSs, particularly when the latter are derived from well-validated and adequately powered models.

Conversely, GRSs and PRSs may better capture the polygenic architecture of complex metabolic diseases but differ substantially across studies in terms of variant selection, the number of variants included, weighting methods, and validation strategies. Scores ranged from models based on a limited number of susceptibility variants to genome-wide approaches incorporating thousands or even millions of SNPs, potentially capturing different dimensions of genetic susceptibility and further limiting direct comparability across studies [33,185,186,187,189]. This lack of standardization currently limits comparability across investigations and may contribute to discrepant findings. In addition, most studies included in this review were conducted in populations of predominantly European ancestry [17,28,29,151,156,162,177,182,183,185,188]. Because allele frequencies, linkage disequilibrium patterns, and variant effect sizes may differ across populations, both candidate-gene associations and GRS/PRS performance may not be directly transferable to other ancestral groups. Consequently, greater emphasis on multi-ancestry studies and external validation is needed to improve the generalizability and clinical relevance of nutrigenetic findings.

Another important source of heterogeneity relates to the definition and assessment of MedDiet adherence. Across studies, adherence was quantified using different dietary indices and scoring systems, including the MDS [162,185,187,189], modified Mediterranean diet scores [182], the 14-item PREDIMED adherence questionnaire [28,155,177], and other adherence-based measures [163,164]. Mediterranean dietary interventions also varied substantially, ranging from MedDiet supplemented with extra virgin olive oil or nuts in the PREDIMED trial [17,28,29,156] to a personalized hypocaloric MedDiet in patients with MASLD [183]. In addition, several studies compared Mediterranean dietary patterns with low-fat diets [30,151,174,184], whereas others investigated specific components of the Mediterranean dietary pattern, particularly MUFA intake and dietary fat quality [157,158,160,178,179]. Further variability arises from differences in the timing of dietary assessment. While some studies evaluated MedDiet adherence only at baseline [162,177,185,189], others assessed dietary exposure during follow-up or through structured dietary interventions [17,28,29,156,183,184]. As dietary habits may change over time, baseline measures may not accurately reflect long-term dietary exposure. Likewise, even in intervention studies, variations in adherence and concurrent dietary changes cannot be completely excluded. Consequently, studies categorized under the broad definition of “Mediterranean diet” may be evaluating substantially different dietary exposures, further complicating comparisons across investigations and the interpretation of gene–diet interactions. As a result, the apparent replication of a statistically significant gene–diet interaction across studies should be interpreted with caution, since the underlying dietary exposure may differ substantially in its composition, assessment method, timing of evaluation, or nutritional targets.

Importantly, associations between MedDiet adherence and favorable metabolic outcomes should not be interpreted as evidence of a gene–diet interaction. A true interaction implies that the magnitude or direction of the dietary effect differs according to genetic background. However, several studies included in this review reported independent associations of both MedDiet adherence and genetic susceptibility with disease-related outcomes, without demonstrating a statistically significant interaction between the two factors [28,29,177,183,184,185,188,189]. Distinguishing between independent associations, effect modification, and formal gene–diet interactions is therefore essential when interpreting the available literature. Moreover, causal inference remains limited in much of the available literature. A substantial proportion of studies relied on observational designs, which are prone to residual confounding and measurement error, particularly in dietary assessment [162,163,164,177,182,185,188]. Although some intervention studies have been conducted, their number remains limited, and several had relatively small sample sizes [30,157,158,161,184]. Detecting gene–diet interactions generally requires substantially larger sample sizes than those needed to identify main genetic or dietary effects. Therefore, limited statistical power may have hindered the detection of modest interaction effects, particularly in dietary intervention trials. Consequently, borderline or non-significant interaction terms should be interpreted cautiously, particularly in studies with limited statistical power, and may warrant further investigation in adequately powered populations.

An additional consideration is the degree of independent replication underlying apparently consistent findings. A substantial proportion of the candidate-gene evidence derives from Mediterranean research populations, particularly from PREDIMED, CORDIOPREV, and related cohorts or intervention settings. Although these studies have generated important and biologically relevant evidence, findings arising from the same or closely related populations cannot be considered equivalent to independent replication in external populations. Conversely, some genetic associations have been investigated across different study designs or populations, providing a stronger basis for reproducibility. Overall, the extent of independent replication remains limited for many reported gene–diet interactions, and apparently consistent findings should therefore be interpreted in light of the degree of overlap in study populations and cohorts. Replication in independent, ancestrally diverse populations will be essential to establish the robustness and generalizability of these findings.

Multiple testing represents an additional concern, particularly in candidate-gene studies evaluating multiple variants, genetic models, dietary exposures, and outcomes. Accordingly, exploratory interaction findings should be interpreted cautiously, particularly when they have not been independently replicated.

A further consideration concerns the interpretation of statistically significant gene–diet interactions. Genetic association refers to a relationship between a genetic variant or measure of genetic susceptibility and a phenotype or disease outcome, whereas a statistical gene–diet interaction indicates that the magnitude or direction of the dietary association differs according to genetic background. However, a statistically significant interaction does not by itself demonstrate a biological or causal interaction at the molecular level. Establishing a biological mechanism requires additional evidence, such as functional studies, experimental validation, or mechanistic evidence linking the genetic variant, dietary exposure, and relevant biological pathway. Accordingly, the gene–diet interactions identified in the studies included in this review should primarily be interpreted as statistical evidence of effect modification unless supported by independent biological or experimental evidence.

Moreover, dietary intake was frequently assessed using self-reported dietary assessment instruments, including food-frequency questionnaires and diet-adherence scores, which are inherently subject to recall bias, reporting inaccuracies, and measurement error. Capturing long-term dietary exposure is particularly challenging, as dietary habits may fluctuate over time and may not be adequately reflected by a single assessment.

Despite these limitations, available data consistently support a beneficial role of Mediterranean dietary patterns across metabolic diseases. In contrast, evidence for clinically relevant gene–diet interactions remains less robust and more heterogeneous. Although some studies have reported interactions between MedDiet adherence and genetic susceptibility, findings remain inconsistent across diseases, genetic models, and outcomes. Consequently, current knowledge remains insufficient to justify the routine implementation of genetically tailored MedDiet recommendations in clinical practice. The limited consistency of current findings suggests that genetic susceptibility alone is unlikely to fully explain the considerable interindividual variability observed in response to Mediterranean dietary patterns. This complexity is further underscored by the rapidly evolving therapeutic landscape of metabolic diseases, including the emergence of GLP-1 receptor agonists for obesity and T2D and the recent introduction of resmetirom for MASLD [190,191]. Future studies should therefore investigate how genetic susceptibility, dietary patterns, and pharmacological interventions jointly contribute to interindividual variability in metabolic outcomes. Beyond addressing current methodological limitations, future research should move towards a more comprehensive characterization of the biological mechanisms underlying interindividual responses to the MedDiet. Although most studies have focused on genetic variants and aggregate measures of genetic susceptibility, genetic background represents only one component of the complex network of factors influencing metabolic responses to dietary exposures.

Integrative multi-omics approaches may help clarify the biological pathways linking diet, genetic susceptibility, and metabolic health. In addition to genomic information and PRSs, transcriptomic, epigenomic, metabolomic, lipidomic, and proteomic data may provide complementary insights into gene regulation, metabolic responses, and intermediate biological pathways associated with dietary exposure and disease risk. The gut microbiome may represent an additional mediator of dietary effects and could partly explain interindividual variability in response to Mediterranean dietary patterns.

Artificial intelligence and machine-learning approaches may further facilitate the integration of high-dimensional genomic, multi-omics, dietary, and clinical data, potentially enabling the identification of complex patterns of interindividual response that may not be captured by conventional statistical models. However, their application will require large, well-characterized datasets, external validation, and transparent and interpretable models before they can support clinically actionable precision-nutrition strategies.

Ultimately, integrating genomic, epigenomic, transcriptomic, metabolomic, lipidomic, proteomic, microbiome, and clinical data may provide a more comprehensive framework for precision nutrition than genetic information alone. Such approaches could contribute to the identification of biologically meaningful patient subgroups and potentially improve the personalization of dietary recommendations for the prevention and management of metabolic diseases.

## 7. Limitations

This review has some limitations that should be acknowledged. First, given the broad and multidisciplinary scope of the review, the literature search was not intended to be exhaustive and was based primarily on PubMed; therefore, the selection and interpretation of studies may be subject to selection and reporting bias, and some relevant studies indexed exclusively in other bibliographic databases may not have been identified. Second, the evidence discussed encompasses studies with substantial heterogeneity in study populations, genetic backgrounds, phenotypic definitions, analytical approaches, and clinical endpoints, which limits direct comparisons across studies. In addition, much of the available evidence, particularly regarding genetic associations and polygenic scores, is derived from observational or associative analyses and does not necessarily establish causality. Finally, this is a rapidly evolving field, and emerging genomic approaches, increasingly diverse study populations, and advances in precision medicine may substantially expand or modify the current evidence base. These considerations should be taken into account when interpreting the findings and perspectives presented in this review.

## 8. Conclusions

The available literature consistently supports a beneficial role of the MedDiet across metabolic diseases, while evidence that genetic background substantially modifies these effects remains limited and heterogeneous. Although genetic susceptibility may contribute to interindividual differences in dietary response, statistically significant gene–diet interactions have been demonstrated only in a subset of studies and outcomes. Therefore, the current knowledge is insufficient to support the routine implementation of genetically tailored MedDiet recommendations in clinical practice.

Future studies should prioritize larger multi-ancestry populations, standardized dietary assessment methods, and integrative multi-omics approaches incorporating genetic, molecular, and microbiome data. These efforts may provide a more comprehensive understanding of the biological mechanisms underlying interindividual variability in dietary responses and help advance precision nutrition in the prevention and management of metabolic diseases.

## Figures and Tables

**Table 1:** Genetic scores in metabolic diseases: current evidence and potential applications.

Reference	Disease	Genetic Score	Main Findings	Potential Application
Ashenhurst et al., 2022 [139]	T2D	T2D PRS	Earlier diagnosis, incident T2D	Risk prediction
Corella et al., 2016 [140]	T2D	Obesity-specific GRS: nobGRS (5 SNPs) and obGRS (5 SNPs)	Earlier diagnosis; T2D risk	Risk prediction
Wang et al., 2024 [141]	T2D	Trans-ancestry PRS	T2D risk, age at onset, complications	Risk prediction and complications
Hodgson et al., 2025 [142]	T2D	Pathway-specific scores	Earlier onset, insulin dependence, complications	Disease heterogeneity
Yu et al., 2025 [143]	T2D	Pathway-specific PRSs	Heterogeneity in disease progression and complications	Disease heterogeneity
Khera et al., 2019 [145]	Obesity	Genome-wide PGS	BMI, severe obesity, cardiometabolic risk	Risk stratification
Smit et al. [146]	Obesity	Multi-ancestry PGS	BMI variance, early-life prediction	Early identification
Xiao et al. [147]	MASLD	MASLD PRS	Liver and cardiometabolic outcomes	Prognostic stratification
Jamialahmadi et al. [148]	MASLD	Partitioned PRSs	Distinct MASLD phenotypes	Phenotype characterization
Shirono et al. [149]	MASLD	Two-variant PRSs	Prognostic heterogeneity by obesity	Prognostic assessment
Kim et al. [138]	Multi-trait	O-MetPRS/ D-MetPRS	Multimorbidity, GLP-1 treatment	Integrated metabolic risk

Abbreviations. BMI: body mass index; D-MetPRS: diabetes metabolic PRS; GLP-1: glucagon-like peptide-1; MASLD: metabolic dysfunction-associated steatotic liver disease; nobGRS: non-obese subjects genetic risk score; O-MetPRS: obesity metabolic PRS; obGRS: obese subjects genetic risk score; PGS: polygenic score; PRS: polygenic risk score; SNP: single-nucleotide polymorphism; T2D: type 2 diabetes.

**Table 2:** Candidate-gene studies investigating Mediterranean diet–gene interactions in type 2 diabetes.

Reference	Participants	Design/Duration	Gene/Variant	Dietary Exposure	Main Outcome	Effect Size	Gene–Diet Interaction
Ortega-Azorín et al., 2012 [155]	7052 high cardiovascular risk subjects (Spain: 3430 T2D cases, 3622 controls)	Case–control study (PREDIMED baseline)	*FTO* rs9939609; *MC4R* rs17782313	Mediterranean dietary pattern adherence (14-item score; high vs. low adherence)	T2D	Low MedDiet adherence: *FTO* risk allele OR = 1.21 (95%CI: 1.03–1.40); *MC4R* risk allele OR = 1.17 (95%CI: 1.01–1.36). High adherence: associations disappeared.	*FTO* rs9939609 × MedDiet adherence: *p*-interaction = 0.039; *MC4R* rs17782313 × MedDiet adherence: *p* = 0.009; aggregate genetic score × MedDiet adherence: *p* = 0.006. High adherence to the MedDiet counteracted genetic predisposition to T2D
Corella et al., 2013 [156]	7018 high cardiovascular risk subjects (Spain)	PREDIMED RCT; 4.8 years	*TCF7L2* rs7903146	Mediterranean dietary pattern (adherence score and MedDiet intervention with EVOO or nuts vs. control diet)	T2D, fasting glucose, plasma lipids, stroke incidence	TT vs. CC: T2D OR = 1.87 (95%CI: 1.62–2.17). Low MedDiet adherence: higher fasting glucose in TT carriers (132.3 ± 3.5 vs. 127.3 ± 3.2 mg/dL).	*TCF7L2* rs7903146 × MedDiet adherence: no significant interaction for T2D prevalence (*p* = 0.251); fasting glucose (*p* = 0.004), total cholesterol (*p* = 0.005), LDL-C (*p* = 0.003), TG (*p* = 0.046).
Lai et al., 2025 [30]	35 overweight/obese adults (United States: 21 CC, 14 TT)	Randomized crossover trial; 1-week low-fat diet and 1-week MetDiet separated by washout	*TCF7L2* rs7903146	Mediterranean dietary pattern (MedDiet vs. low-fat diet intervention)	TRLs, VLDL subclasses	MedDiet: lower triglyceride-rich lipoprotein and VLDL subclass concentrations than LF diet. Under low-fat diet, TT carriers: higher XS-VLDL concentrations than CC carriers (*p* = 0.032). Under MedDiet, no genotype-related differences in TRL profile.	*TCF7L2* rs7903146 × diet: no significant interaction for TRL changes (*p* > 0.05). Less favorable lipoprotein profile in TT carriers after the low-fat diet; attenuation of genotype-related differences following the MedDiet, although no significant gene–diet interaction was observed.
Pérez-Martínez et al., 2005 [157]	59 healthy adults (Spain: 42 GG, 17 GA)	Randomized crossover dietary intervention; three 4-week dietary periods	SCARB1 rs4238001	Single dietary components (SFA-rich, MUFA-rich [olive oil], and high-carbohydrate diets)	Insulin sensitivity (SSPG), NEFA	MUFA-rich diet: lower SSPG values in GA vs. GG carriers (greater insulin sensitivity; *p* = 0.030). Lower NEFA concentrations in A-allele carriers across all dietary interventions.	*SCARB1* rs4238001 × dietary fat quality: effect observed after MUFA-rich diet but not after SFA-rich or high-carbohydrate diets
Gómez et al., 2005 [158]	59 healthy young adults (Spain: 39 CC, 20 CT/TT)	Randomized crossover dietary intervention; three 4-week dietary periods	LIPC −514 C/T	Mediterranean dietary pattern and comparator diets (MedDiet, SFA-rich diet, and high-carbohydrate diet)	Insulin sensitivity (SSPG), FFA	Male T-allele carriers: higher SSPG concentrations (lower insulin sensitivity) after the SFA-rich diet than after the Mediterranean or high-carbohydrate diets. No significant differences across diets in CC carriers.	LIPC −514 C/T × dietary fat quality: improvement in insulin sensitivity in male T-allele carriers after Mediterranean and high-carbohydrate diets compared with the SFA-rich diet; no effect observed in women
Corella et al., 2016 [17]	7098 high-cardiovascular-risk participants (Spain)	PREDIMED RCT; median follow-up 4.8 years	*CLOCK* rs4580704	Mediterranean dietary pattern intervention (MedDiet supplemented with EVOO or nuts vs. low-fat control diet)	T2D incidence	G-allele carriers: lower T2D incidence than CC homozygotes (HR = 0.69, 95%CI: 0.54–0.87; *p* = 0.002).	*CLOCK* rs4580704 × MedDiet intervention: stronger protective association of the G allele against T2D in the MedDiet group (HR = 0.58, 95%CI 0.43–0.78; *p* < 0.001) than in the control group (HR = 0.95, 95%CI 0.63–1.44; *p* = 0.818); interaction *p* = 0.052

Abbreviations: EVOO: extra virgin olive oil; FFA: free fatty acids; HOMA-IR: Homeostasis Model Assessment-Insulin Resistance; HR: hazard ratio; LDL-C: low-density lipoprotein cholesterol; MedDiet: Mediterranean diet; MUFA: monounsaturated fatty acid; NEFA: non-esterified fatty acid; RCT: randomized controlled trial; SFA: saturated fatty acid; SSPG: steady-state plasma glucose; T2D: type 2 diabetes; TRLs: triglyceride-rich lipoproteins; VLDL: very-low-density lipoprotein.

**Table 3:** Candidate-gene studies investigating Mediterranean diet–gene interactions in obesity and adiposity.

Reference	Participants	Design/Duration	Gene/Variant	Dietary Exposure	Main Outcome	Effect Size	Gene–Diet Interaction
Razquin et al., 2010 [29]	776 high cardiovascular risk subjects (Spain)	PREDIMED RCT; 3-year follow-up	*FTO* rs9939609	Mediterranean dietary pattern intervention (MedDiet supplemented with VOO or nuts vs. low-fat diet)	Body weight change	Baseline: AA genotype, highest body weight. A-allele carriers (AA + AT) vs. TT, lower body weight gain after 3 years (β = −0.685; *p* = 0.022). MedDiet groups: A-allele carriers vs. TT, lower body weight gain (β = −0.830; *p* = 0.018).	*FTO* rs9939609 × MedDiet: not significant (*p* = 0.649; additive model *p* = 0.660).
Corella et al., 2012 [28]	7052 high cardiovascular risk subjects (Spain)	Cross-sectional study (PREDIMED baseline)	*FTO* rs9939609; *MC4R* rs17782313	Mediterranean dietary pattern adherence score (0–14 points)	BMI, WC, obesity	FTO rs9939609 associated with higher BMI (β = 0.17 kg/m^2^ per A allele; *p* = 0.007), WC (β = 0.53 cm per A allele; *p* = 0.003), and obesity (OR = 1.10, 95%CI: 1.02–1.17; *p* = 0.014). Aggregate genetic score: 7% increase in obesity odds per risk allele (OR = 1.07, 95%CI: 1.01–1.13)	AdMedDiet × rs9939609: not significant (BMI *p* = 0.614; WC *p* = 0.229). AdMedDiet × aggregate score: BMI *p* = 0.844; WC *p* = 0.981. Higher MedDiet adherence associated with attenuation of obesity-related genetic susceptibility, although no statistically significant gene–diet interaction was observed.
Di Renzo et al., 2018 [161]	188 subjects (139 assigned to the MedDiet, 49 controls, Italy)	RCT; 4-week intervention	*FTO* rs9939609	Mediterranean dietary pattern intervention (MedDiet vs. control group)	Body composition (total body fat, gynoid fat, TBW)	MedDiet: greater reduction in total body fat (*p* = 0.00) and gynoid body fat (*p* = 0.04). FTO rs9939609: association with TBW variation (*p* = 0.02).	Total body fat (kg): rs9939609 × MedDiet (*p* = 0.04). A-allele carriers: greater total body fat reduction in the MedDiet group and greater total body fat gain in controls. Gynoid body fat: not significant (*p* = 0.32). Total body water: not significant (*p* = 0.47).
Roswall et al., 2014 [162]	11,048 adults (five European countries)	Case–cohort study (DiOGenes); median follow-up 6.8 years.	*FTO* rs9939609; *TCF7L2* rs7903146	Mediterranean dietary pattern adherence (MDS)	Weight change, WC, WC adjusted for BMI.	Higher MDS: lower WC change (β = −0.010 cm/year, *p* = 0.03), lower WC adjusted for BMI change (β = −0.008 cm/year, *p* = 0.02), lower risk of extreme weight gain (OR = 0.98, 95%CI: 0.96–1.00; *p* = 0.04); no association with weight change (*p* = 0.53).	*TCF7L2* rs7903146 × MDS: borderline significant for weight change (*p* = 0.05); beneficial MDS effect in carriers of 1–2 T risk alleles. *FTO* rs9939609 × MDS: not significant.
Sotos-Prieto [163]	1120 adults (Puerto Rico)	Cross-sectional study (Boston Puerto Rican Health Study)	TCF7L2 rs7903146, rs12255372	Mediterranean dietary pattern adherence (high vs. low)	BMI, weight, WC	rs7903146 T-carriers with high MedDiet: lower weight (*p* = 0.013) and WC (*p* = 0.021) vs. CC. rs12255372 T-carriers with high MedDiet: lower anthropometric measures (trend only)	*TCF7L2* rs7903146 × MedDiet adherence: BMI (*p* = 0.060), weight (*p* = 0.024), WC (*p* = 0.026); rs12255372 × MedDiet adherence: BMI (*p* = 0.036), weight (*p* = 0.034), WC not significant (*p* = 0.132)
Aoun et al. [164]	392 adults (Mediterranean region)	Cross-sectional study	*FTO* rs9939609, *MC4R* rs17782313, *MTHFR* rs1801133	Mediterranean dietary pattern adherence (MedDiet Scale, MedDiet Score)	BMI, WC, WHR	*FTO*: higher WHR in AA carriers (*p* = 0.036); *MC4R*: higher WC (*p* = 0.027) and WHR (*p* = 0.007) in CC carriers; *MTHFR*: higher WHR in T-allele carriers (*p* = 0.050)	*FTO* rs9939609, *MC4R* rs17782313,* MTHFR* rs1801133 × MedDiet adherence: no evidence of a protective effect; higher MedDiet adherence did not attenuate obesity-related traits among genetically predisposed individuals.
Razquin et al., 2010 [168]	737 high cardiovascular risk subjects (Spain)	PREDIMED RCT; 3-year follow-up	*IL6* rs1800795	Mediterranean dietary pattern intervention (MedDiet supplemented with VOO, Mediterranean diet supplemented with nuts, or low-fat diet)	Body weight change, BMI	Baseline: CC genotype associated with higher body weight (*p* = 0.024) and BMI (*p* = 0.020). MedDiet + VOO: CC vs. GG + GC, lower body weight gain (β = −2.061; *p* = 0.003)	IL6 rs1800795 × MedDiet + VOO: *p* = 0.028. Lowest weight gain in CC carriers assigned to MedDiet + VOO

Abbreviations: AdMedDiet: adherence to Mediterranean Diet; BMI: body mass index; MDS: Mediterranean diet score; MedDiet: Mediterranean diet; RCT: randomized controlled trial; TBW: total body water; VOO: virgin olive oil; WC: waist circumference; WHR: waist-to-hip ratio.

**Table 4:** Candidate-gene studies investigating Mediterranean diet–gene interactions in metabolic syndrome.

Reference	Participants	Design/Duration	Gene/Variant	Dietary Exposure	Main Outcome	Effect Size	Gene–Diet Interaction
Ortega-Azorín et al. [169]	7166 high-cardiovascular-risk participants (Spain)	PREDIMED RCT; median follow-up 4.8 years	*MLXIPL* rs3812316	Mediterranean dietary pattern intervention (MedDiet vs. low-fat diet) and baseline Mediterranean dietary pattern adherence	TG, hypertriglyceridemia, myocardial infarction	G-allele carriers: lower TG (β = −0.109 mmol/L; *p* < 0.001); lower hypertriglyceridemia risk (OR = 0.73; *p* = 1.4 × 10^−6^). MedDiet intervention: lower TG during follow-up (*p* = 0.030).	*MLXIPL* rs3812316 × MedDiet adherence: hypertriglyceridemia (*p*-interaction = 0.025); high Mediterranean diet adherence, OR = 0.63 (*p* = 8.6 × 10^−6^) vs. low adherence, OR = 0.88 (*p* = 0.219). *MLXIPL* rs3812316 × MedDiet intervention: myocardial infarction HR = 0.34 (*p* = 0.036) in the MedDiet group vs. HR = 0.90 (*p* = 0.830) in the control group.
Gomez-Delgado et al., 2014 [172]	507 patients with coronary heart disease (Spain)	CORDIOPREV RCT; 12 months	*TNF-α*: rs1800629 (−308 G>A); rs1799964	Mediterranean dietary pattern intervention (MedDiet vs. low-fat diet)	TG, hsCRP, postprandial TG response, glucose metabolism	At baseline, rs1800629 GG genotype: higher TG (*p* = 0.003), ApoB (*p* = 0.01), hsCRP (*p* = 0.003), and postprandial TG response (*p* = 0.025) vs. A-allele carriers.	*TNF* rs1800629 × MedDiet: TG (*p* = 0.005), hsCRP (*p* = 0.034); baseline genotype differences abolished after MedDiet intervention. *TNF* rs1799964 × MedDiet: not significant
Garcia-Rios et al., 2018 [174]	424 patients with coronary heart disease (Spain)	CORDIOPREV RCT; 12 months	*CETP* rs3764261	Mediterranean dietary pattern intervention (MedDiet vs. low-fat diet)	HDL-C; TG	After MedDiet, T-allele carriers (TT + TG): higher HDL-C (*p* = 0.021) and lower TG (*p* = 0.020) than GG homozygotes; no genotype differences after low-fat diet.	*CETP* rs3764261 × dietary pattern (MedDiet vs. low-fat diet): HDL-C (*p* = 0.006), TG (*p* = 0.040).
Coltell et al., 2021 [177]	954 high-cardiovascular-risk participants (Spain)	Cross-sectional study (PREDIMED baseline)	*ADIPOQ* variants: rs17300539; rs2241766; rs1501299; rs17366568	Mediterranean dietary pattern adherence (14-item score)	Adiponectin, HDL-C, TG, fasting glucose, T2D	Adiponectin positively associated with HDL-C (*p* = 6.6 × 10^−36^) and inversely associated with triglycerides (*p* = 4.7 × 10^−18^), fasting glucose (*p* = 3.5 × 10^−16^), and T2D (*p* = 1.4 × 10^−7^). ADIPOQ rs17300539 associated with higher adiponectin (*p* = 7.2 × 10^−5^).	*ADIPOQ* rs17300539 × MetDiet: not significant (*p* = 0.559); rs2241766 × MedDiet: not significant (*p* = 0.072); rs1501299 × MedDiet: not significant (*p* = 0.795); rs17366568 × MedDiet: not significant (*p* = 0.525).
AlSaleh et al., 2011 [178]	448 subjects at risk of MetS (366 White Europeans included in gene–diet analyses)	RISCK trial; 24 week-intervention	*ADIPOQ* variants rs17300539; rs182052; rs16861209; rs1501299	Single dietary components/dietary fat composition (high-MUFA diet, low-fat diet, and high-SFA reference diet)	Serum adiponectin, insulin sensitivity	rs1501299 T allele: higher adiponectin (*p* = 0.006); rs182052 A allele: lower adiponectin (*p* = 0.03). After high-MUFA diet, rs182052 GG: +3.8% adiponectin vs. GA + AA: −2.6% (*p* = 0.006).	ADIPOQ rs182052 × high-MUFA diet: adiponectin (*p* = 0.006); rs182052 × age × diet: adiponectin (*p* = 0.07); rs1501299 × diet: not significant.
de Luis et al., 2019 [179]	363 obese adults (Spain)	RCT; 12 weeks	*ADIPOQ* rs1501299	Single dietary components/dietary fat composition (hypocaloric MUFA-rich diet vs. PUFA-rich diet)	Adiponectin, insulin, HOMA-IR	Both diets: decreased body weight, BMI, fat mass, waist circumference, systolic blood pressure, and leptin levels. MUFA diet: lower total cholesterol and LDL-C in both genotypes.	*ADIPOQ* rs1501299 × dietary fatty acids. Only GG carriers showing increased adiponectin and improved insulin resistance after both diets. PUFA diet: insulin (*p* = 0.01) and HOMA-IR (*p* = 0.02) for GG vs. GT + TT. MUFA diet: insulin (*p* = 0.01) and HOMA-IR (*p* = 0.03).
Hajiluian et al., 2018 [180]	200 adults (150 patients with MetS + 50 controls, Iran)	Case–control study	*VEGF* rs2010963	Mediterranean dietary pattern adherence (Med-DQI)	Anthropometric and metabolic parameters	Higher dietary cholesterol score: higher TG (*p* = 0.04). Lower fish intake: higher LDL-C (*p* = 0.02). Lower fruit/vegetable intake: higher LDL-C (*p* = 0.05).	Genotype-specific differences in MedDQI components observed; however, no formal gene–diet interaction analysis reported.
Garaulet et al., 2009 [160]	1100 adults (United States)	Cross-sectional study (GOLDN)	*CLOCK*: rs4580704, rs1801260 (3111T/C)	Single dietary components (MUFA intake and SFA intake)	Glucose, HOMA-IR, waist circumference	rs4580704 G-allele carriers associated with lower insulin (*p* = 0.016), lower systolic blood pressure (*p* = 0.001), lower diastolic blood pressure (*p* = 0.005), and lower hypertension risk. rs1801260 GG genotype associated with higher BMI (*p* = 0.017), insulin (*p* = 0.043), and HOMA-IR (*p* = 0.009)	rs4580704 × MUFA (>13.2% energy): glucose *p* = 0.020; HOMA-IR *p* = 0.026. rs1801260 × SFA (>11.8% energy): waist circumference *p* = 0.017.
Garcia-Rios et al., 2014 [151]	475 patients with MetS (Spain)	CORDIOPREV RCT; 12 months	*CLOCK* rs1801260	Mediterranean dietary pattern intervention (MedDiet vs. low-fat diet)	Insulin, HOMA-IR, QUICKI	Low-fat diet: TT vs. TC + CC, lower insulin (*p* = 0.032), lower HOMA-IR (*p* = 0.027), higher QUICKI (*p* = 0.024). No significant genotype differences after MedDiet. No significant effects on lipid traits	*CLOCK* rs1801260 × dietary pattern: insulin concentrations (*p* = 0.009), HOMA-IR (*p* = 0.014), QUICKI (*p* = 0.028). Low-fat diet: in TT homozygotes, lower insulin concentrations (*p* = 0.032), lower HOMA-IR (*p* = 0.027), and higher QUICKI (*p* = 0.024) than C-allele carriers. MedDiet: not significant

Abbreviations: HDL-C: high-density lipoprotein cholesterol; HOMA-IR: Homeostatic Model Assessment of Insulin Resistance; hsCRP: high-sensitivity C-reactive protein; LDL-C: low-density lipoprotein cholesterol; MedDiet: Mediterranean diet; MedDQI: Mediterranean Dietary Quality Index; MetS: metabolic syndrome; MUFA: monounsaturated fatty acids; OR: odds ratio; QUICKI: quantitative insulin sensitivity check index; PUFA: polyunsaturated fatty acids; SFA: saturated fatty acids; TG: triglycerides.

**Table 5:** Candidate-gene studies investigating Mediterranean diet–gene interactions in metabolic dysfunction-associated steatotic liver disease.

Reference	Participants	Design/Duration	Gene/Variant	Dietary Exposure	Main Outcome	Effect Size	Gene–Diet Interaction
Chen et al., 2024 [182]	21,619 participants (UK Biobank)	Population-based cohort study	*PNPLA3* rs738409; *TM6SF2* rs58542926	Mediterranean dietary pattern adherence (MMED) and specific dietary components (fruit/vegetable/legume intake, fish intake, and red/processed meat intake)	LFC, cT1 (fibrosis/inflammation), liver-related outcomes	Higher MMED: lower LFC (β = −0.30% per quartile; *p* = 3.66× 10^−29^); higher fruit/vegetable/legume intake: LFC (β = −0.42%; *p* = 4.95 × 10^−12^); higher fish intake: lower LFCt (β = −0.44%; *p* = 4.11 × 10^−10^); higher red/processed meat intake: higher liver fat content (β = 0.47%; *p* = 3.01 × 10^−11^)	*PNPLA3* rs738409 × MMED (*p* = 0.029) and fruit/vegetable/legume intake (*p* = 0.018); *TM6SF2* rs58542926 × MMED (*p* = 0.013), fruit/vegetable/legume intake (*p* = 0.016), and red/processed meat intake (*p* = 0.029). Dietary effects on LFC: up to 3.8-fold higher in PNPLA3 GG vs. CC carriers
Troisi et al., 2026 [183]	148 adults with MASLD (78 completed 6-month follow-up, Italy)	Prospective cohort study; 6 months	*PNPLA3* rs738409, *TM6SF2* rs58542926, *MBOAT7* rs641738, *GCKR* rs1260326	Mediterranean dietary pattern intervention (personalized hypocaloric MedDiet)	CAP, liver stiffness, metabolic and metabolomic response	Mean weight loss ≈8% and CAP reduction ≈17% after intervention; steatosis improvement increased with greater weight loss	Greater CAP reduction observed among *PNPLA3* risk-allele carriers following the intervention; however, no formal gene–diet interaction analysis reported.
Dogay et al., 2025 [184]	250 adults with MASLD and BMI ≥25 kg/m^2^ (Turkey)	RCT; 12 weeks	*PNPLA3* rs738409	Mediterranean dietary pattern intervention (hypocaloric MedDiet vs. low-fat diet)	CAP, LSM, body weight	Both diets: lower CAP (β = −45.80 dB/m, *p* < 0.001), lower LSM (β = −1.06 kPa, *p* < 0.001), lower body weight (β = −4.46 kg, *p* < 0.001); no difference between MedDiet and low-fat diet for CAP (*p* = 0.976), LSM (*p* = 0.355), or weight (*p* = 0.159)	*PNPLA3* rs738409 × diet: not significant for CAP (*p* = 0.286), LSM (*p* = 0.464), and body weight (*p* = 0.622). No significant group × genotype × time interaction for CAP (*p* = 0.449).

Abbreviations: CAP: Controlled Attenuation Parameter; cT1: iron-controlled T1 time; LFC: liver fat content; LSM: liver stiffness measurement; MedDiet: Mediterranean diet; MMED: modified Mediterranean diet score; RCT: randomized controlled trial.

**Table 6:** Genetic risk score studies evaluating Mediterranean diet interactions in metabolic diseases.

Reference	Participants	Design/Duration	Gene/Variant	Dietary Exposure	Main Outcome	Effect Size	Gene–Diet Interaction
Langenberg et al., 2014 [185]	28,557 adults, Europe (12,403 incident T2D cases and 16,154 subcohort participants, Europe)	Prospective cohort Study (EPIC-InterAct); mean 11.7 years	T2D GRS (49 established T2D loci)	Mediterranean dietary pattern adherence (MDS: low, medium, and high adherence)	Incident T2D	Per 1-SD increase in GRS: HR = 1.41 (95%CI: 1.34–1.49) for T2D	No significant interaction between the GRS and MDS for incident T2D (*p* > 0.1). Benefits of MedDiet adherence observed irrespective of genetic risk level.
Hardy et al., 2026 [186]	9488 adults (8283 European Americans; 1205 African Americans)	Longitudinal cohort study	Ancestry-specific T2D PRS (224 SNPs in European Americans; 22 SNPs in African Americans)	Dietary pattern adherence (Mediterranean, DASH, and Southern dietary patterns)	Incident T2D	High-risk PRS, highest vs. lowest tertile: OR = 1.24 (95%CI: 1.06–1.45) in European Americans and OR = 1.61 (95%CI: 1.22–2.12) in African Americans. MedDiet, highest vs. lowest tertile: OR = 0.68 (95%CI: 0.53–0.87) for incident T2D in African Americans.	MedDiet adherence and PRS independently associated with T2D risk; no formal gene–diet interaction estimate reported.
Hosseini-Esfahani et al., 2017 [187]	1254 adults (627 obesity cases and 627 matched controls, Iran)	Nested case–control study (Tehran Lipid and Glucose Study)	Weighted *FTO*-GRS based on 6 variants: rs1421085, rs1121980, rs17817449, rs8050136, rs9939973, rs3751812	Mediterranean dietary pattern adherence (quartiles of MDS)	Obesity, abdominal obesity, WHR	High GRS (≥6): obesity OR across increasing MDS quartiles = 1.00, 0.80, 0.79, 0.67 (*p*-trend = 0.001). Low GRS (<6): OR = 1.33, 1.06, 0.97, 1.12 (*p*-trend = 0.33).	*FTO*-GRS × MDS: obesity (*p* = 0.04); abdominal obesity (*p* = 0.29); high WHR (*p* = 0.37). Protective association of higher MedDiet adherence evident only among participants with higher genetic risk.
Baratali et al., 2021 [188]	2542 adults (Switzerland)	Prospective cohort study (CoLaus cohort); follow-up 5.3 years	Weighted obesity GRS based on 31 and 68 obesity-associated SNPs	Dietary pattern adherence (two Mediterranean Diet Scores, AHEI, and other dietary patterns)	Weight, BMI, waist and hip circumference changes	Higher adherence to MedDiet scores associated with lower risk of weight gain > 5 kg and WC increase >5 cm.	No significant interactions between obesity GRSs and MedDiet scores. Dietary effects largely independent of genetic susceptibility.
Seral-Cortes et al., 2020 [32]	605 adolescents (Europe)	Cross-sectional study (HELENA)	Obesity-GRS based on 21 obesity-associated SNPs	Mediterranean dietary pattern adherence (MDS, 0–9)	BMI, WC, MetS score	MedDiet adherence inversely associated with BMI (males: *p* = 0.008; females: *p* < 0.001), WC (males: *p* = 0.030; females: *p* < 0.001), and MetS score (males: *p* = 0.047; females: *p* = 0.002)	Obesity-GRS × MedDiet interaction for BMI (males: *p* = 0.003; females: *p* < 0.001), WC (males: *p* = 0.009; females: *p* < 0.001), and MetS score (males: *p* = 0.014; females: *p* = 0.006). Stronger interaction effects in females.
Seral-Cortes et al., 2025 [33]	1982 children at baseline, 1649 at FU1, 1907 at FU2; 1254 participants in longitudinal analyses, Europe)	Cross-sectional analyses at baseline, 2-year follow-up, and 6-year follow-up; longitudinal analyses over 6 years (IDEFICS/I.Family Study	BMI-PRS based on 2,100,302 variants	Mediterranean dietary pattern adherence (MDS) and specific Mediterranean diet components (individual food groups)	BMI, WC	No significant association between MDS and adiposity measures in adjusted longitudinal models. Higher vegetable and legume intake associated with lower BMI (β = −0.01, 95%CI: −0.02 to 0.00) and WC (β = −0.02, 95%CI: −0.03 to 0.00) at the 6-year follow-up.	Significant BMI-PRS × MDS interaction for BMI at baseline (β = 0.12, 95%CI: 0.01–0.24) and at the 6-year follow-up (β = −0.19, 95%CI −0.38 to −0.01); no significant interaction for WC or in longitudinal analyses.
San-Cristobal et al., 2017 [189]	1263 adults (Europe)	Web-based RCT (Food4Me study); 6 months	Multi-trait GRS based on 14 SNPs related to MetS traits	Mediterranean dietary pattern adherence (MDS)	BMI, WC, glucose, total cholesterol, carotenoids, omega-3 index	Higher baseline MDS associated with greater reductions in BMI (*p* = 0.011), WC (*p* = 0.010), and glucose concentrations (*p* = 0.022) during the intervention. Lower GRS associated with greater reduction in total cholesterol than higher GRS (*p* = 0.043)	Significant GRS × MDS interaction observed only for circulating carotenoids (*p* = 0.006). No significant interactions for BMI (*p* = 0.689), WC (*p* = 0.226), glucose (*p* = 0.718), total cholesterol (*p* = 0.567), or omega-3 index (*p* = 0.209).
Chen et al., 2024 [182]	21,619 participants (UK Biobank)	Population-based cohort study	16-variant hepatic steatosis PRS	Mediterranean dietary pattern adherence (MMED)	LFC, cT1 (fibro-sis/inflammation),	Higher MMED associated with lower LFC (β per MMED quartile = −0.30%, 95%CI −0.35 to −0.25; *p* = 3.66 × 10^−29^). Higher PRS associated with higher LFC (β per SD = 0.53%, 95%CI 0.47–0.60; *p* = 2.65 × 10^−63^)	Significant PRS × MMED interaction for LFC (interaction β = −0.07, *p* = 0.012). The protective effect of the MedDiet was stronger in the highest vs. lowest PRS quartile (β = −0.43 vs. −0.25 per MMED quartile; *p* for heterogeneity = 0.014). Similar interactions were observed for cT1.
Troisi et al., 2026 [183]	148 adults with MASLD (78 completed 6-month follow-up, Italy)	Prospective cohort study; 6 months	Weighted PRS based on *PNPLA3*, *TM6SF2*, *MBOAT7* and *GCKR*	Mediterranean dietary pattern intervention (personalized hypocaloric MedDiet)	CAP, liver stiffness, metabolomic response	Significant reductions in body weight, CAP, glycemic parameters, lipids, and liver enzymes after the intervention.	No significant association was observed between the dichotomized PRS and CAP reduction following dietary intervention.

Abbreviations: AHEI: Alternative Healthy Eating Index; BMI: body mass index; CAP: Controlled Attenuation Parameter; cT1: iron-controlled T1 time; DASH: Dietary Approaches to Stop High Blood Pressure; FU1: 1st follow-up (2 years); FU2: 2nd follow-up (6 years); GRS: genetic risk score; HR: hazard ratio; LFC: liver fat content; MDS: Mediterranean diet score; MedDiet: Mediterranean diet; MetS: metabolic syndrome; MMED: modified Mediterranean diet score; OR: odds ratio; PRS: polygenic risk score; SD: standard deviation; SNP: single-nucleotide polymorphism; T2D: type 2 diabetes; WC: waist circumference; WHR: waist-to-hip ratio.

**Table 7:** Qualitative synthesis of the evidence for gene–diet interactions across metabolic diseases.

Disease	Candidate-Gene Evidence	GRS/PRS Evidence	Overall Evidence
T2D	Moderate, heterogeneous	Limited; no formal interaction demonstrated	Moderate–limited
Obesity	Moderate, heterogeneous; several significant interactions but limited replication	Moderate, heterogeneous; significant interactions in some studies but inconsistent findings	Moderate
MetS	Moderate, heterogeneous; several formal interactions but limited replication and heterogeneous dietary exposures	Limited; no consistent evidence of interaction	Moderate–limited
MASLD	Limited–moderate; strong signal from a large population-based study but limited replication	Limited–moderate; promising signal from a large population-based study but limited replication	Limited–moderate

Strength of evidence was assessed qualitatively based on the consistency of findings, presence of formal statistical interaction, study design, sample size, and replication across studies. This qualitative classification was not intended as a formal risk-of-bias assessment or evidence-grading framework. Abbreviations: GRS: genetic risk score; MASLD: metabolic dysfunction-associated steatotic liver disease; MetS: metabolic syndrome; PRS: polygenic risk score; T2D: type 2 diabetes.

## Data Availability

No new data were created or analyzed in this study. Data sharing is not applicable to this article.

## Abbreviations

The following abbreviations are used in this manuscript:

BMI	Body mass index
CVD	Cardiovascular disease
GRS	Genetic risk score
GWAS	Genome-wide association study
HDL	High-density lipoproteins
LFC	Liver fat content
LDL	Low-density lipoproteins
MASLD	Metabolic dysfunction-associated steatotic liver disease
MedDiet	Mediterranean diet
MetS	Metabolic syndrome
MUFA	Monounsaturated fatty acids
PGS	Polygenic score
PRS	Polygenic risk score
SNP	Single-nucleotide polymorphism
T2D	Type 2 diabetes
TG	Triglycerides
VLDL	Very-low-density lipoproteins
WC	Waist circumference
WHR	Waist-to-hip ratio
